# Pyrimidine depletion enhances targeted and immune therapy combinations in acute myeloid leukemia

**DOI:** 10.1172/jci.insight.173646

**Published:** 2024-04-22

**Authors:** Ola A. Elgamal, Sydney Fobare, Sandip Vibhute, Abeera Mehmood, Dennis C. Vroom, Mariah L. Johnson, Blaise Stearns, James R. Lerma, Jean Truxall, Emily Stahl, Bridget Carmichael, Shelley J. Orwick, Alice S. Mims, Emily Curran, Ramasamy Santhanam, Susheela Tridandapani, Mitch A. Phelps, Zhiliang Xie, Christopher C. Coss, Sharyn D. Baker, Jeffrey Patrick, Janel K. Ezzell, Jayesh Rai, Jianmin Pan, Shesh N. Rai, Cody Stillwell, Mark Wunderlich, Mouad Abdulrahim, Thomas E. Goodwin, Gerard Hilinski, Chad E. Bennett, Erin Hertlein, John C. Byrd

**Affiliations:** 1 Division of Hematology and Oncology, Department of Internal Medicine, College of Medicine, University of Cincinnati, Cincinnati, Ohio, USA.; 2 Division of Hematology, Department of Internal Medicine, College of Medicine;; 3 Medicinal Chemistry Shared Resource, Comprehensive Cancer Center;; 4 College of Pharmacy and Comprehensive Cancer Center; and; 5 Drug Development Institute, Comprehensive Cancer Center, The Ohio State University, Columbus, Ohio, USA.; 6 Division of Biostatistics and Bioinformatics, Department of Environmental and Public Health Sciences, College of Medicine;; 7 Cancer Data Science Center, College of Medicine; and; 8 Biostatistics and Informatics Shared Resource, University of Cincinnati Cancer Center, University of Cincinnati, Cincinnati, Ohio, USA.; 9 Division of Experimental Hematology and Cancer Biology, Cancer and Blood Diseases Institute, Cincinnati Children’s Hospital Medical Center, Cincinnati, Ohio, USA.; 10 Department of Chemistry, Hendrix College, Conway, Arkansas, USA.

**Keywords:** Oncology, Therapeutics, Cancer immunotherapy, Drug therapy, Leukemias

## Abstract

Acute myeloid leukemia (AML) is a fatal disease characterized by the accumulation of undifferentiated myeloblasts, and agents that promote differentiation have been effective in this disease but are not curative. Dihydroorotate dehydrogenase inhibitors (DHODHi) have the ability to promote AML differentiation and target aberrant malignant myelopoiesis. We introduce HOSU-53, a DHODHi with significant monotherapy activity, which is further enhanced when combined with other standard-of-care therapeutics. We further discovered that DHODHi modulated surface expression of CD38 and CD47, prompting the evaluation of HOSU-53 combined with anti-CD38 and anti-CD47 therapies, where we identified a compelling curative potential in an aggressive AML model with CD47 targeting. Finally, we explored using plasma dihydroorotate (DHO) levels to monitor HOSU-53 safety and found that the level of DHO accumulation could predict HOSU-53 intolerability, suggesting the clinical use of plasma DHO to determine safe DHODHi doses. Collectively, our data support the clinical translation of HOSU-53 in AML, particularly to augment immune therapies. Potent DHODHi to date have been limited by their therapeutic index; however, we introduce pharmacodynamic monitoring to predict tolerability while preserving antitumor activity. We additionally suggest that DHODHi is effective at lower doses with select immune therapies, widening the therapeutic index.

## Abstract 2

Dihydroorotate dehydrogenase inhibitor HOSU-53 shows efficacy in acute myeloid leukemia and promotes long-term survival in mouse models when combined with anti-CD47 therapy.

## Introduction

Drug development in hematologic cancers historically consisted of broad DNA-damaging therapeutics that often mediate genotoxic damage and have a narrow therapeutic window but has recently migrated toward targeted and immune therapeutics with improved safety profiles. In adults, this is exemplified best in chronic myeloid leukemia, chronic lymphocytic leukemia, non-Hodgkin’s lymphoma, Hodgkin’s disease, and multiple myeloma, where several new targeted or immune therapies have significantly impacted outcome. For all these diseases, the frequency of initial and salvage allogeneic stem cell transplant has decreased, reflective of development of more effective and tolerable therapies that have improved survival.

Acute myeloid leukemia (AML) represents the most commonly diagnosed and lethal adult leukemia, with a 5-year survival of less than 15% in patients over the age of 60 (1, 2). AML originates from a leukemia-initiating hematopoietic stem cell and is characterized by uncontrolled proliferation of dysfunctional, immature hematopoietic myeloid cells (blasts). AML-initiating cells have features that resemble normal hematopoietic stem cells and are highly immune suppressive, making it quite challenging to treat with either targeted or immune-based therapies (outside of allogeneic stem cell transplant). Until recently, effective therapies for AML were limited to intensive chemotherapy followed by allogeneic stem cell transplant, which provided curative potential for a minority of patients. More recently, several therapies targeting select mutations, including IDH1 (ivosidenib) (3), IDH2 (enasidenib) (4), and FLT3 (gilteritinib) (5), have been approved for AML treatment and demonstrate a unique differentiating phenotype. By differentiating early AML progenitor cells, these therapies may further separate their phenotype from normal hematopoietic cells and in some cases can promote durable remissions. However, these targeted therapeutics are limited to AML patients with IDH1, IDH2, or FLT3 mutations, and resistance mechanisms have been reported (6, 7). Another example of mutation-specific, targeted differentiation therapy is all-*trans* retinoic acid (ATRA) for acute promyelocytic leukemia (APL) AML subtype. Despite the high response rate, there is a lack of durable response in most patients in the absence of combination therapy (8–10). This collective experience suggests that successful therapy will involve differentiation agents that can be used in a broader AML population and identifying combination strategies likely to result in durable remissions. The need for combination strategies is supported by murine model data of AML, where a combination of differentiation and targeted therapies is required to eliminate clonal AML stem cells (11). Broadly affecting AML with differentiation therapy will require a therapeutic target that is effective in AML subtypes independent of mutation subtype.

One such target for broad differentiation of AML cells demonstrated by Sykes and Scadden et al. (12) and later corroborated by others is dihydroorotate dehydrogenase (DHODH) (13–15). DHODH is a mitochondrial membrane flavoprotein that catalyzes the rate-limiting step of the de novo pyrimidine nucleotide biosynthesis pathway (16, 17). DHODH catalyzes the oxidation of dihydroorotate (DHO) to orotate, which is further converted downstream by uridine monophosphate synthetase to uridine monophosphate (18, 19), the biosynthetic precursor of thymidine and cytidine (20). Rapidly proliferating cells and most cancer cells rely predominantly on this de novo synthesis pathway (20) as opposed to the salvage pathway, which re-cycles degraded intracellular nucleic acids (17) or shuttles extracellular nucleosides using nucleoside transporters (12, 17, 20). Thus, inhibiting de novo pyrimidine synthesis would interfere with numerous metabolic and anabolic cell survival processes, explaining the cytotoxic response of tumor cells to DHODH inhibitors (DHODHi). In AML, the differentiating effect of DHODHi has recently been attributed to inducing replicative stress, an expected response of pyrimidine depletion (21–24).

The earlier clinical application of a relatively potent DHODHi, such as brequinar (BRQ), in solid tumor malignancies was halted because of lack of efficacy and significant dose-limiting stomatitis, gastrointestinal toxicity, and hematologic toxicity (25, 26). This contrasts with less potent DHODHi, such as leflunomide and teriflunomide, that are used to treat rheumatoid arthritis and multiple sclerosis and lack these toxicities associated with BRQ. Notably, in a phase I clinical study, leflunomide showed significant activity in multiple myeloma, achieving stable disease in 9/11 patients (27), corroborating a high translational impact for novel and more potent DHODHi. While BRQ and other potent DHODHi have advanced as monotherapies in early clinical trials targeting hematological malignancies, including AML (13, 14, 28), we hypothesized that effective use of a potent DHODHi would require mechanistic combination therapies, which build upon the features of differentiation and strategies to predict tolerable doses. Herein, we describe the preclinical characterization of a selective and potent DHODHi, HOSU-53, for AML treatment and the use of pharmacodynamic monitoring of plasma DHO levels as a method to determine safe HOSU-53 doses. We also identify multiple combination strategies with HOSU-53, including a mechanistically unrecognized pathway of enhancing innate immune checkpoint inhibitor therapy in myeloid malignancies.

## Results

### HOSU-53 is a potent and selective inhibitor of DHODH enzyme.

**Figure 1:**
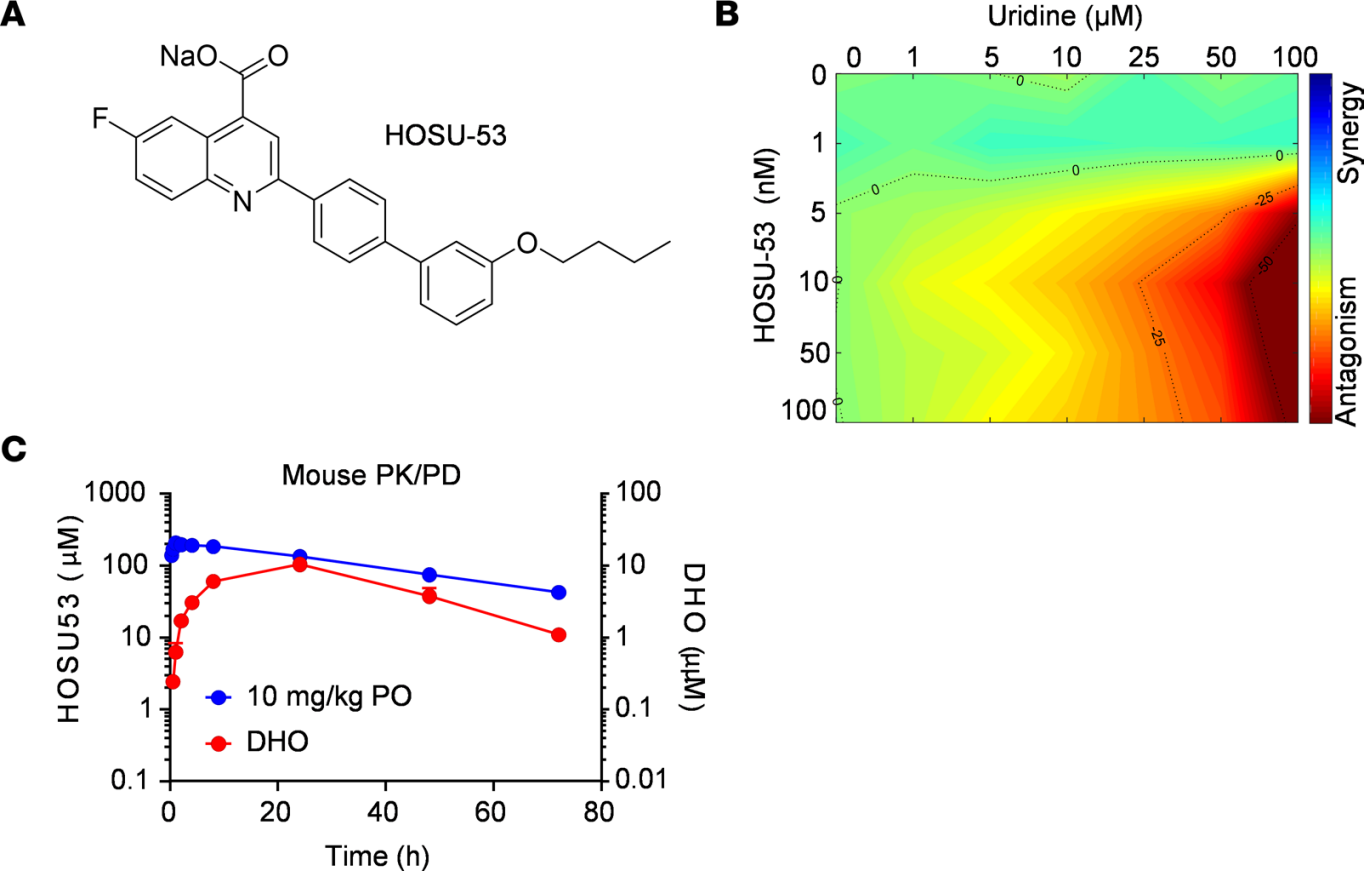
HOSU-53 is a selective DHODHi. (**A**) Chemical structure of HOSU-53. (**B**) In vitro uridine rescue assay using MOLM-13 AML cell line. Combenefit software was used to evaluate highest single agent synergy and antagonism between increasing concentrations of HOSU-53 and exogenous uridine (*n* = 3). (**C**) In vivo pharmacokinetics (PK) and pharmacodynamics (PD) modeling after single-dose administration of intravenous (i.v.) or oral (p.o.) HOSU-53 in wild-type male mice (*n* = 3/group). Data shown as mean and error SD.

We generated a series of chemically distinct, selective DHODHi (Hendrix College-Ohio State University [HOSU-number] compounds) and identified HOSU-53 as our lead clinical candidate molecule (Figure 1A; synthesis details provided in Supplemental Methods; supplemental material available online with this article; https://doi.org/10.1172/jci.insight.173646DS1). HOSU-53 has subnanomolar potency against human DHODH (hDHODH) comparable or superior to other potent clinical DHODHi candidates in cell-free enzyme assays (Table 1). We also compared the potency of HOSU-53 against mouse, rat, and dog DHODH enzyme and observed relatively similar inhibitory potency across the tested species (Supplemental Table 1). The synthesis of HOSU-53 is quite simple using the Suzuki reaction (29), making the generation of therapeutically relevant quantities of HOSU-53 feasible.

We confirmed HOSU-53 selectivity to the de novo pyrimidine synthesis pathway via supplementing exogenous uridine (12), demonstrating the on-target emergence of metabolic resistance to HOSU-53 activity by increasing uridine concentration above 25 μM (Figure 1B). This is well above the uridine concentration in AML patient bone marrow and blood plasma, which ranged from 0.35 to 2.16 μM (average 1.14 μM) in bone marrow and 0.3 to 1.04 μM (average 0.67 μM) in blood plasma (Table 2). This suggests that uridine levels observed in patients would be insufficient to overcome the activity of HOSU-53. We additionally verified lack of alternative-target activity at 10 μM against a panel of approximately 450 human kinases (Supplemental Figure 1).

To verify in vivo bioavailability and on-target activity, we conducted a pharmacokinetic (PK) and pharmacodynamic (PD) study administering a single intravenous (i.v.) or oral (p.o.) dose of HOSU-53 in male C57BL/6 mice. This study demonstrated favorable oral bioavailability (85%) and a terminal half-life of 29 hours. We noted accumulation of DHO, the upstream substrate of DHODH, in the plasma, verifying on-target activity and illustrating DHO as a clinically practical plasma pharmacodynamic biomarker (Figure 1C and Table 3), as recently reported by others (14). We also verified favorable pharmaceutical properties, including in vitro absorption, distribution, metabolism, and excretion, as well as impressive PK in rats and dogs (Supplemental Table 2). Together, our data support the selectivity and potency of HOSU-53 as a DHODHi, prompting further preclinical evaluation of HOSU-53 in AML.

### HOSU-53 exhibits potent in vitro antileukemic activity.

**Figure 2:**
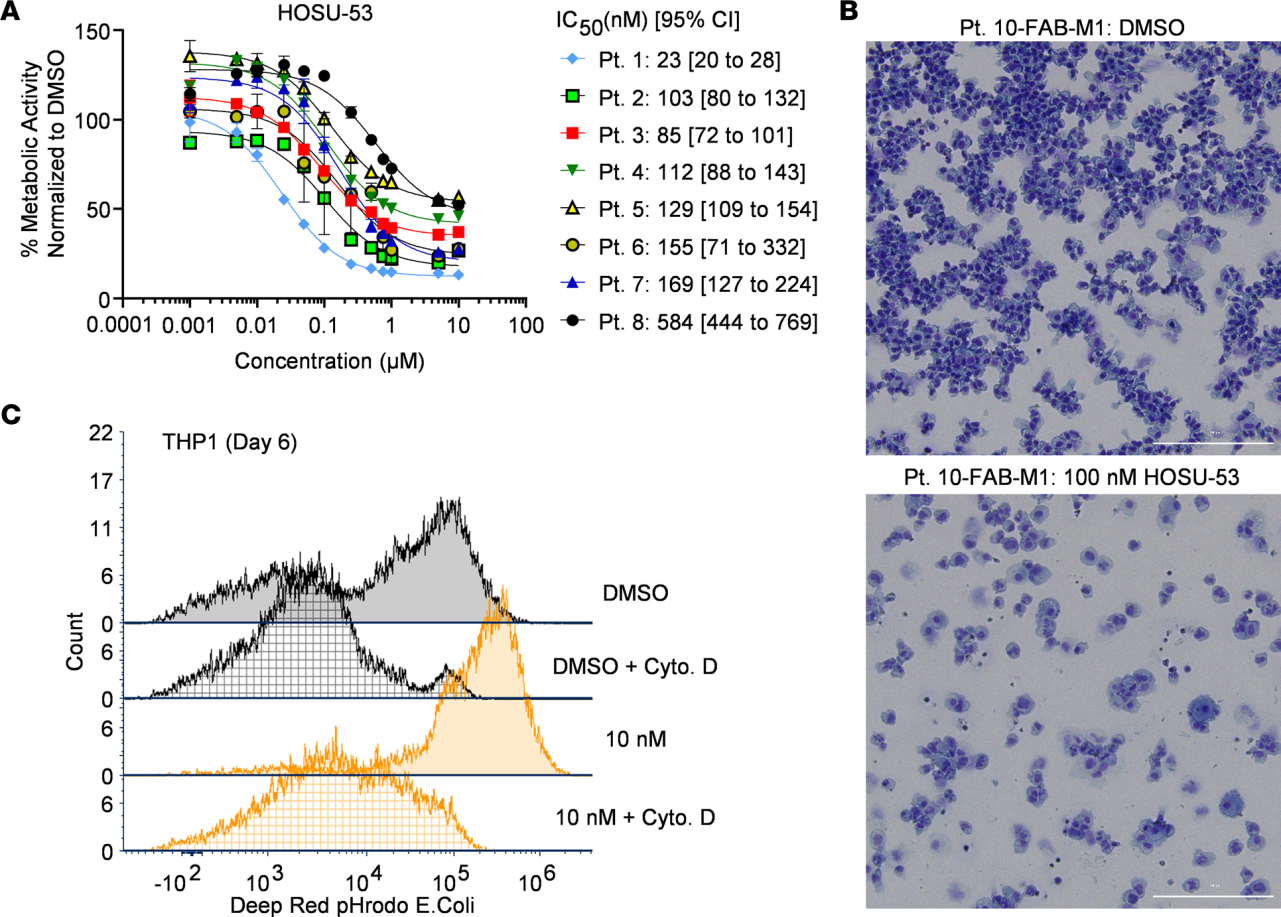
HOSU-53 demonstrates in vitro potency and differentiation properties in AML. (**A**) MTS proliferation assay using primary AML samples (*n* = 8) to determine the 50% inhibitory concentration (IC_50_) of HOSU-53 after 96-hour treatment. Data shown as mean and error SD. GraphPad Prism was used to analyze, visualize, and calculate IC_50_ values. (**B**) Representative hema 3 differential staining (Thermo Fisher Scientific) of cytospin preparation slides for a primary AML sample treated with HOSU-53 in vitro for 7 days (long-term culture, LTC) to determine morphology changes. Images were taken using BioTek Cytation 5 Cell Imaging Multimode Reader at 20× original magnification. (**C**) Representative flow cytometry histogram plot for in vitro *E*. *coli* phagocytosis assay using THP-1 cells treated with HOSU-53 for 6 days. Cytochalasin D (Cyto. D), an actin polymerization inhibitor was used as an inhibitor of phagocytosis. Phagocytosis assay experiments were done 3 independent times.

We first evaluated the in vitro potency of HOSU-53 in multiple AML cell lines and observed superiority to BRQ (Supplemental Table 3). We next sought to characterize the effect of HOSU-53 on primary AML patient samples (sample characteristics provided in Supplemental Table 4). HOSU-53 exhibited an antileukemic profile with nanomolar potency across multiple AML samples with a median IC_50_ of 120.5 nM (Figure 2A) and reduced cell viability (Supplemental Figure 2A). Furthermore, HOSU-53 induced a differentiated morphology (Figure 2B and Supplemental Figure 2B) and increased CD11b^+^CD14^+^ cells (Supplemental Figure 2C), suggesting these cells could resume innate immune cell function. In addition to morphology changes, another surrogate marker of differentiation is restoration of phagocytic immune function typical of mature macrophages (30). We found that HOSU-53–treated THP-1 cells exhibited enhanced ability to phagocytose *E*. *coli* (Figure 2C), suggesting that HOSU-53 promotes in vitro innate phagocytic function.

### HOSU-53 demonstrates in vivo single-agent antileukemic activity.

**Figure 3:**
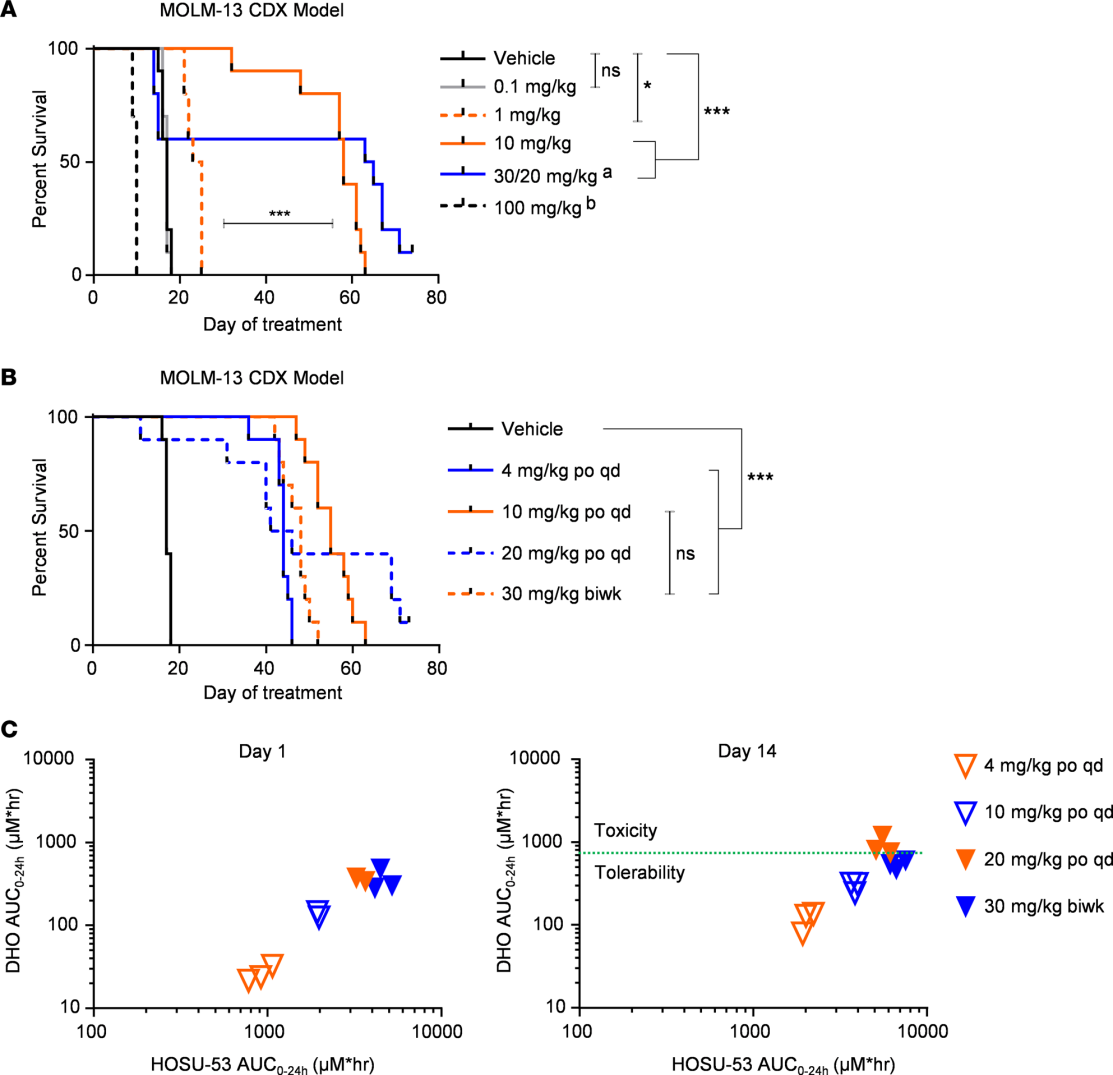
The use of DHO plasma concentration to gauge HOSU-53 tolerability and toxicity. (**A**) Multiple ascending dose study to determine the maximum in vivo daily tolerated oral dose of HOSU-53. Using the FLT3-mutant MOLM-13 CDX tumor–bearing model, 4 days after i.v. engraftment, NCG mice (*n* = 10/group) were enrolled to receive increasing HOSU-53 daily p.o. doses. ^a^Mice in the 30 mg/kg group showed weight loss by day 13 of treatment. Thus, a dosing holiday was instituted followed by resuming treatment on day 18 at 20 mg/kg reduced dosage. ^b^Mice in the 100 mg/kg groups showed weight loss instituting a dosing halt on day 6 of treatment. Acceptable tolerability was defined as a group mean body weight (BW) loss of less than 20% during the study. Any dosing regimen resulting in greater BW loss was considered above the maximum tolerated dose (MTD). Adjusted FDR *P* value *<0.05, ***≤0.0001. (**B** and **C**) Correlative efficacy and PK/PD in vivo analysis of HOSU-53. Using the FLT3-mutant MOLM-13 CDX tumor–bearing model, 4 days after i.v. engraftment, NCG mice (*n* = 10/group for survival study, *n* = 3/group for PK/PD analysis) were enrolled to receive increasing HOSU-53 oral doses to correlate efficacy (**B**) with the plasma concentration of HOSU-53 (PK) and on-target metabolite, DHO accumulation (PD) at day 1 and cumulative day 14 (**C**). Green dotted line represents the hypothesized threshold of tolerability. Data represent individual values of each mouse. Adjusted FDR *P* value ***≤0.0001. qd, daily; biwk, twice weekly.

To evaluate the efficacy of HOSU-53 in AML, we first determined the maximum tolerated dose (MTD) using the MOLM-13 cell line–derived xenograft (CDX) disseminated AML model (31). After treating mice with escalating doses of daily oral HOSU-53 ranging from 0.1 to 100 mg/kg, we determined that as low as daily 1 mg/kg achieved a modest yet significant survival advantage (Figure 3A; *P* value < 0.05). However, 10 mg/kg was well tolerated and had improved prolonged survival (*P* value < 0.0001). Daily 30 mg/kg induced dose-limiting weight loss after 13 days of treatment. A brief dosing holiday allowed the mice to recover, and dosing was reinitiated at 20 mg/kg in the remaining mice and was tolerated for more than 50 additional days. We found that daily administration of 100 mg/kg was toxic, resulting in rapid weight loss, discontinuation of dosing at day 6, and mice meeting early removal criteria (ERC) by day 10. These data suggest that the MTD in mice is a daily dose between 10 and 20 mg/kg.

### DHO plasma level correlates with in vivo tolerability and toxicity.

Next, we performed a follow-up MOLM-13 study to determine the correlation between efficacy and the plasma levels of HOSU-53 and DHO following daily oral administration of 4, 10, and 20 mg/kg HOSU-53 compared with twice weekly 30 mg/kg. The 20 mg/kg dose was intolerable in approximately half the mice yet was tolerated well by the other half (Figure 3B), reinforcing that the MTD is between 10 and 20 mg/kg daily. Nonetheless, 30 mg/kg twice weekly was well tolerated and provided a similar survival advantage as daily 10 mg/kg (median survival of 48 days versus 55 days, respectively; adjusted FDR *P* value 0.39), suggesting that intermittent dosing of DHODHi can still be efficacious. We measured the plasma concentrations of HOSU-53 and DHO at day 1 and day 14 of cumulative treatment and observed a linear exposure-response relationship between HOSU-53 plasma area under the 24-hour concentration-time curve (AUC_0–24_) and DHO AUC_0–24_ (Figure 3C). Collectively, our efficacy study paired with PK/PD analysis indicate that 10 mg/kg is an efficacious and tolerable daily regimen, higher doses can be safely administered intermittently, and DHO accumulation serves as an important biomarker to identify in vivo therapeutic responses and toxicity.

### HOSU-53 demonstrates superior preclinical in vivo activity.

**Figure 4:**
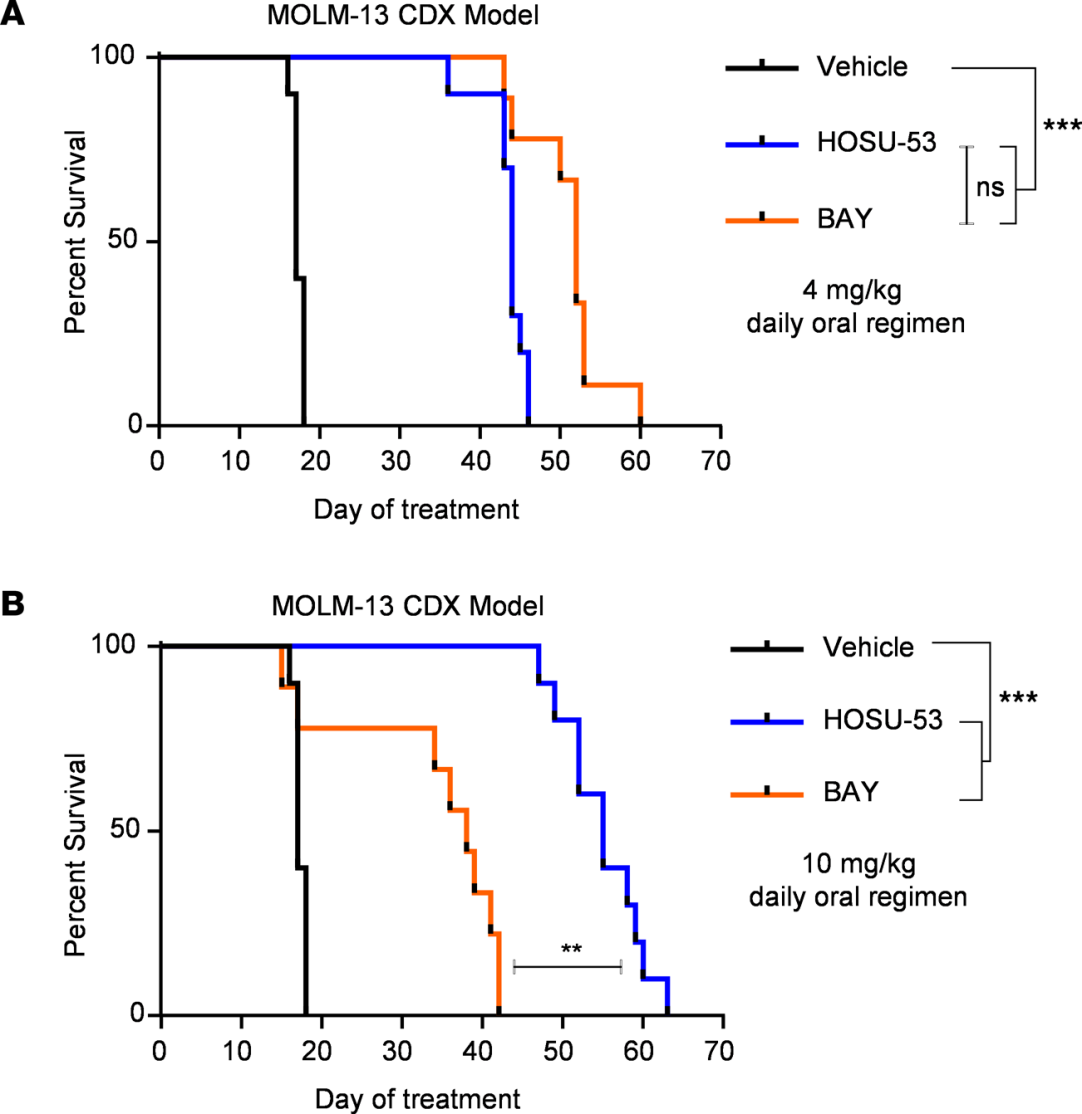
HOSU-53 results in superior in vivo outcome using AML disseminated xenograft model. (**A**) Using the FLT3-mutant MOLM-13 CDX tumor–bearing model, 4 days after i.v. engraftment, NCG mice (*n* = 10/group) were enrolled to receive daily p.o. 4 mg/kg of HOSU-53 or BAY to compare with (**B**) daily p.o. 10 mg/kg of HOSU-53 or BAY and determine using dose-matched regimens the overall survival after HOSU-53 versus BAY, a potent clinical DHODHi candidate. In the 4 mg/kg and 10 mg/kg BAY groups, 1/10 mice were excluded from analysis because of non-treatment-related accidents: gavage error and animal not found in cage, respectively. Figure 3B and **A** and **B** share the same vehicle and HOSU-53 arms. Adjusted FDR *P* value **≤0.001, ***≤0.0001.

There are several DHODHi candidates that have been pursued for AML whose chemical structures are available, including ASLAN003 (13) and the most potent inhibitor reported to date, BAY (14). BAY had a median IC_50_ of 6.5 nM in primary AML patient samples (Supplemental Figure 3). Thus, we evaluated the efficacy of HOSU-53 and BAY using matching dose levels for both agents and observed that though 4 mg/kg BAY was better than 4 mg/kg HOSU-53, BAY was not tolerated at 10 mg/kg while HOSU-53 at 10 mg/kg significantly improved survival and was still below the identified MTD for HOSU-53 (Figure 4, A and B). Therefore, using a direct comparison of the highest achievable daily dose of HOSU-53 at 10 mg/kg (median survival 55 days) and BAY at 4 mg/kg (median survival 52 days), our study underscores the prominent single-agent in vivo efficacy of HOSU-53.

### HOSU-53 demonstrates in vivo synergy with approved targeted AML therapies.

**Figure 5:**
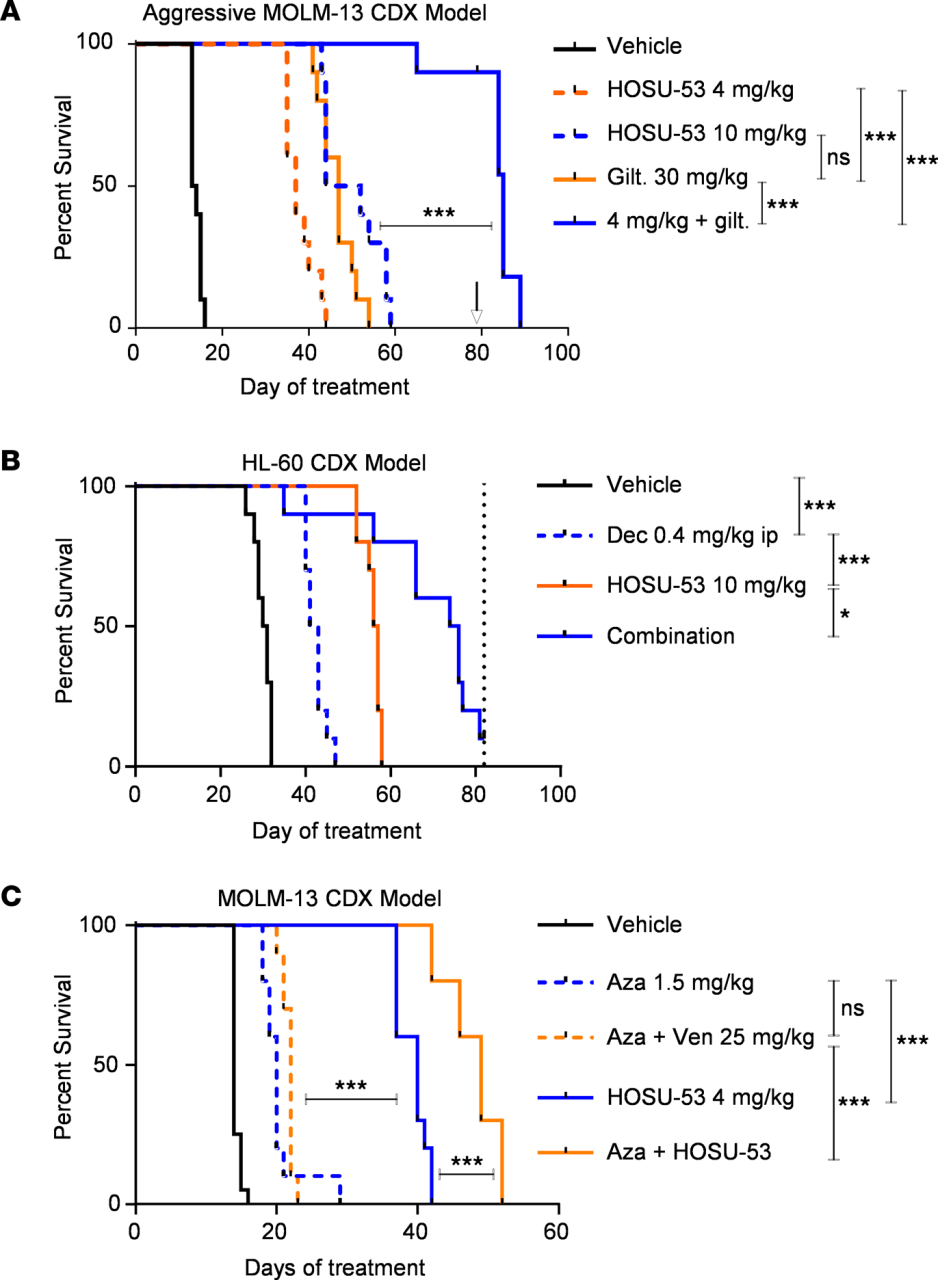
HOSU-53 significantly enhances the outcome of select FDA-approved AML therapies. (**A**) Using the FLT3-mutant MOLM-13 CDX tumor–bearing model, 10 days after i.v. engraftment, NCG mice (*n* = 10/group) were enrolled to receive daily p.o. 4 mg/kg or 10 mg/kg HOSU-53 to compare their efficacy with daily p.o. 30 mg/kg gilteritinib (gilt) FLT3 inhibitor or the combination of 4 mg/kg HOSU-53 with gilt. In the gilt combination cohort, 4 mice were euthanized for tissue harvest to determine disease burden, and the remaining 6 mice were kept on study for survival analysis. Black arrow indicates treatment was stopped at day 79. Adjusted FDR *P* value ***≤0.0001. Vehicle and HOSU-53 arms in this study are shared with Figure 8, A and B. (**B**) Using the P53-null HL-60 CDX tumor–bearing model, 14 days after i.v. engraftment, NCG mice (*n* = 10/group) were enrolled to receive 0.4 mg/kg decitabine (Dec) hypomethylating agent (HMA) i.p. as 4 days on/10 days off cycles or daily p.o. 10 mg/kg HOSU-53 or combination of both agents. Dotted black line indicates treatment was stopped and end of study at day 82. *P* value *≤0.05, ***≤0.0001. (**C**) Using the FLT3-mutant MOLM-13 CDX tumor–bearing model, 4 days after i.v. engraftment, NCG mice (*n* = 10/group) were enrolled to receive daily p.o. 4 mg/kg HOSU-53 to compare its monotherapy efficacy with 1.5 mg/kg azacitidine (aza) HMA i.p. for 5 days every 16 day cycles monotherapy or in combination with daily p.o. 25 mg/kg venetoclax (aza/ven) or daily p.o. 4 mg/kg HOSU-53 (aza/HOSU-53). Adjusted FDR *P* value ***≤0.0001. Supplemental Figure 4 shows all 9 groups done in this study; herein we show the most significant regimens for clarity.

A previous report using CRISPR/Cas9 screening suggested synergy between DHODH and FLT3 inhibition (32). Using these same CRISPR/Cas9 data, another study demonstrated that a switch from glycolysis to oxidative phosphorylation may represent a metabolic adaptation that could mediate gilteritinib (gilt) resistance (33). Therefore, we investigated the synergy between HOSU-53 and gilt using a modified, more aggressive MOLM-13 model in which treatment started 10 days postengraftment (versus 4 days in all other MOLM-13 CDX studies). Although 30 mg/kg gilt was superior to 4 mg/kg HOSU-53 dose, 10 mg/kg HOSU-53 provided a similar median survival (47 versus 48 days). Importantly, 4 mg/kg HOSU-53 in combination with 30 mg/kg gilt demonstrated an enhanced effect, resulting in greater survival (dosing was stopped on day 79; median survival was 84 days), highlighting the advantage offered by the concomitant administration of low-dose HOSU-53 with an FLT3 inhibitor (Figure 5A).

Even with the impressive single-agent activity of HOSU-53, monotherapy strategies are seldom curative owing to the heterogeneity of AML. AMLs with TP53 mutations or deletion are the most challenging to treat (34), and 5 or 10 days of the hypomethylating agent (HMA) decitabine represents one of the standard therapies for TP53-mutated AML (35). However, HMAs like decitabine or azacitidine alone or in combination with the B-cell lymphoma 2 inhibitor venetoclax fail to achieve a long-term response (36, 37). Therefore, we evaluated the efficacy of HOSU-53 alone or in combination with decitabine using a TP53-null HL-60 CDX model. We found that HOSU-53 monotherapy was superior to decitabine, and when combined, these 2 agents were well tolerated and significantly prolonged survival (Figure 5B, decitabine versus combination adjusted FDR *P* value < 0.0001), despite a previous report suggesting a dependence on functional TP53 for DHODHi efficacy (38).

Although azacitidine and venetoclax (aza/ven) is the standard of care for older patients with AML, most patients still relapse and succumb to their disease. Mechanisms of resistance appear to occur via adaptation in fatty acid metabolism and alternative metabolic changes rendering these cells susceptible to mitochondria targeting therapies, such as inhibitors of NADPH, pyruvate dehydrogenase inhibitors, and ClpP protease agonists, that are either effective in this area or add to the therapeutic benefit of this combination (39–41). Given the influence HOSU-53 and other DHODHi may have on mitochondrial function, we compared HOSU-53 efficacy with aza/ven and investigated the benefit of their combination, which we hypothesized would both be tolerable and improve the efficacy of the currently used aza/ven approach. Using the MOLM-13 CDX model, we compared the effect of adding HOSU-53 to either aza or ven versus the aza/ven double approach and explored the outcome of the triple aza/ven/HOSU-53 regimen. In the MOLM-13 CDX model, 25 mg/kg ven monotherapy achieved a modest survival advantage, and efficacy was slightly enhanced when combined with aza (Supplemental Figure 4). HOSU-53 at 4 mg/kg, however, showed superior efficacy to the aza/ven regimen (Figure 5C and Supplemental Figure 4). Adding ven to HOSU-53 did not appear to further enhance survival, though adding aza to HOSU-53 did provide additional benefit (Figure 5C and Supplemental Figure 4). Together, this study indicates the advantage of HOSU-53 monotherapy over the aza/ven regimen and demonstrates the superiority of aza + HOSU-53.

**Figure 6:**
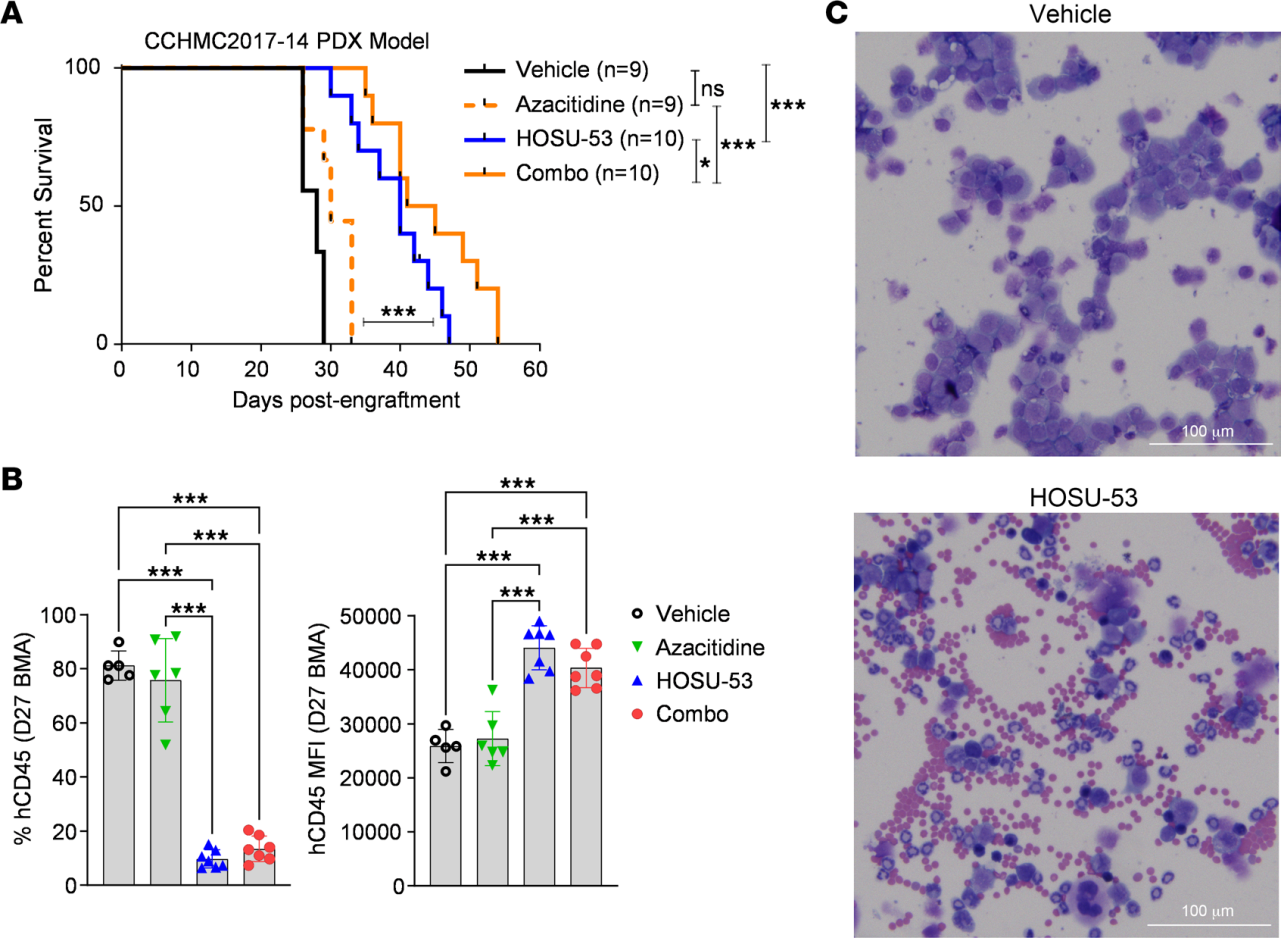
HOSU-53 significantly prolongs survival in a PDX in vivo tumor model. (**A**) Using a relapsed or refractory (r/r) MLL/AF10 rearranged, therapy-related AML passaged PDX sample (CCHMC-2017-14), NRGS mice (*n* = 9–10/group) were intravenously engrafted with 5 × 10^5^ cells/mouse. At 13 days after engraftment, mice were enrolled to receive vehicle, 10 mg/kg HOSU-53 orally 5 days each week, 0.5 mg/kg azacitidine (aza) i.p. 4 days each week for 4 weeks, or combination of aza and HOSU-53. Mice were continually monitored till end removal criteria. (**B**) On day 27 after engraftment (D27), a bone marrow aspirate (BMA) was performed to assess disease burden by measuring the surface expression of human CD45 on live cells (7-AAD–negative cells) using flow cytometry. Data shown as scatter dot plot mean with SD. Adjusted FDR *P* value *<0.05, ***≤0.0001. (**C**) Representative Wright-Giemsa differential staining (Thermo Fisher Scientific) for the D27 BMA cells cytospin preparation slides. Images were taken using BioTek Cytation 5 Cell Imaging Multimode Reader at 40× original magnification.

We further validated HOSU-53 efficacy and synergy with aza in an additional in vivo study using a primary patient-derived xenograft (PDX) model and observed a significant survival advantage with HOSU-53 treatment (Figure 6A). We collected interim bone marrow aspirates (BMAs; 27 days postengraftment and 14 days of treatment) and found a marked reduction in tumor burden indicated by reduced human CD45 (hCD45) percentage in HOSU-53 arms (Figure 6B). Interestingly, we also observed increased CD45 MFI (Figure 6B), suggesting a more mature phenotype (42). Wright-Giemsa staining demonstrated an abundance of mature cells in HOSU-53 cohorts, corroborating the differentiation properties of DHODHi (Figure 6C and Supplemental Figure 5).

### HOSU-53 enhances the efficacy of CD38-directed antibody immunotherapy.

**Figure 7:**
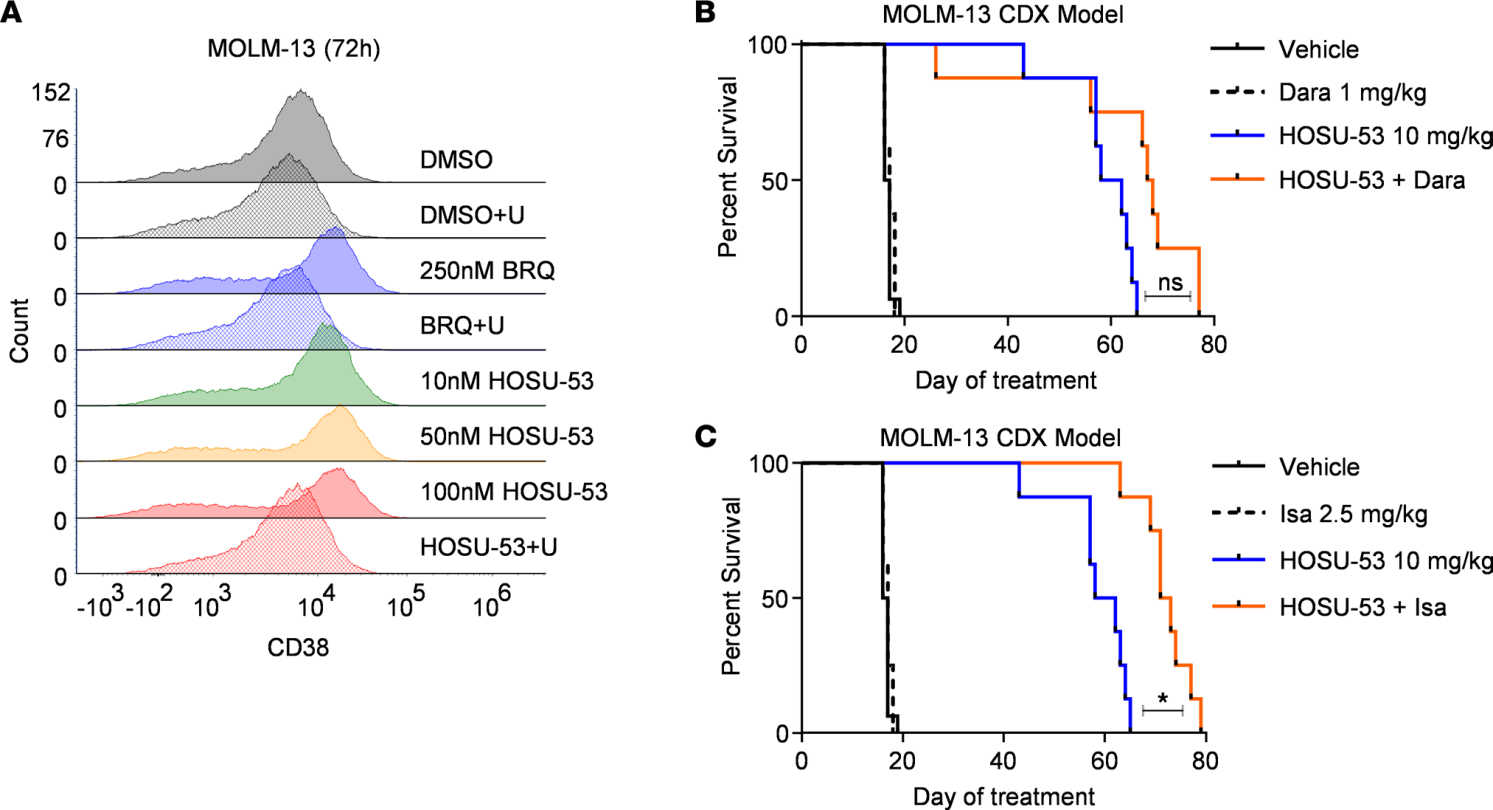
DHODHi mediate the modulation of CD38 surface expression, resulting in synergy with anti-CD38 therapies. (**A**) Representative flow cytometry overlay histogram plot for CD38 surface expression in MOLM-13 cell line following a 72-hour treatment with BRQ or increasing concentrations of HOSU-53 in the presence or absence of 0.1 mM uridine (U) supplementation (experiment was done 3 independent times). (**B** and **C**) Using the FLT3-mutant MOLM-13 CDX tumor–bearing model, 4 days after i.v. engraftment, NCG mice (*n* = 8/group) were enrolled to receive daily p.o. 10 mg/kg HOSU-53 or biwk 1 mg/kg i.p. daratumumab (Dara) (**B**) or biwk 2.5 mg/kg i.p. isatuximab (Isa) (**C**) anti-CD38 antibodies or a combination of HOSU-53 and Dara (**B**) or combination of HOSU-53 and Isa (**C**). **B** and **C** share same vehicle and HOSU-53 monotherapy arms, with data split into 2 figures for clarity. Adjusted FDR *P* value *<0.05.

Our initial characterization of HOSU-53 involved the immunophenotyping of treated primary AML samples, including CD38 as a marker to gate leukemia stem cells (43). Interestingly, we observed a variable increase in CD38 surface expression across AML samples treated with HOSU-53 (Supplemental Figure 6, A and B). To validate this observation, we investigated CD38 modulation in the MOLM-13 AML cell line and verified that CD38 was upregulated by DHODHi. This was inhibited by uridine supplementation, suggesting specificity of this modulation to target the de novo pyrimidine synthesis pathway (Figure 7A). We hypothesized that enhanced surface expression of CD38 mediated by DHODHi could promote enhanced activity with anti-CD38–targeted immunotherapy. These findings bear similarity to Buteyn and colleagues, in which where ATRA-induced CD38 upregulation led to a synergistic response with dara, an anti-CD38 monoclonal antibody therapy (44). Therefore, we used the MOLM-13 CDX model to investigate if HOSU-53 (or BAY) provided synergy with the FDA-approved anti-CD38 therapies, dara and isa. These studies verified that although neither anti-CD38 antibody demonstrated single-agent therapeutic in vivo activity in this model, combination with DHODHi (HOSU-53 or BAY) prolonged overall survival, with the combination of HOSU-53 and isa offering the greatest overall efficacy with 72 days of median survival (Figure 7, B and C, and Supplemental Figure 7, A and B). Our data provide support for future investigation of this combination regimen as part of AML trials and preliminary evidence for the potential synergy between DHODHi and immunotherapy.

### HOSU-53 accentuates the efficacy of CD47-directed antibody immunotherapy.

**Figure 8:**
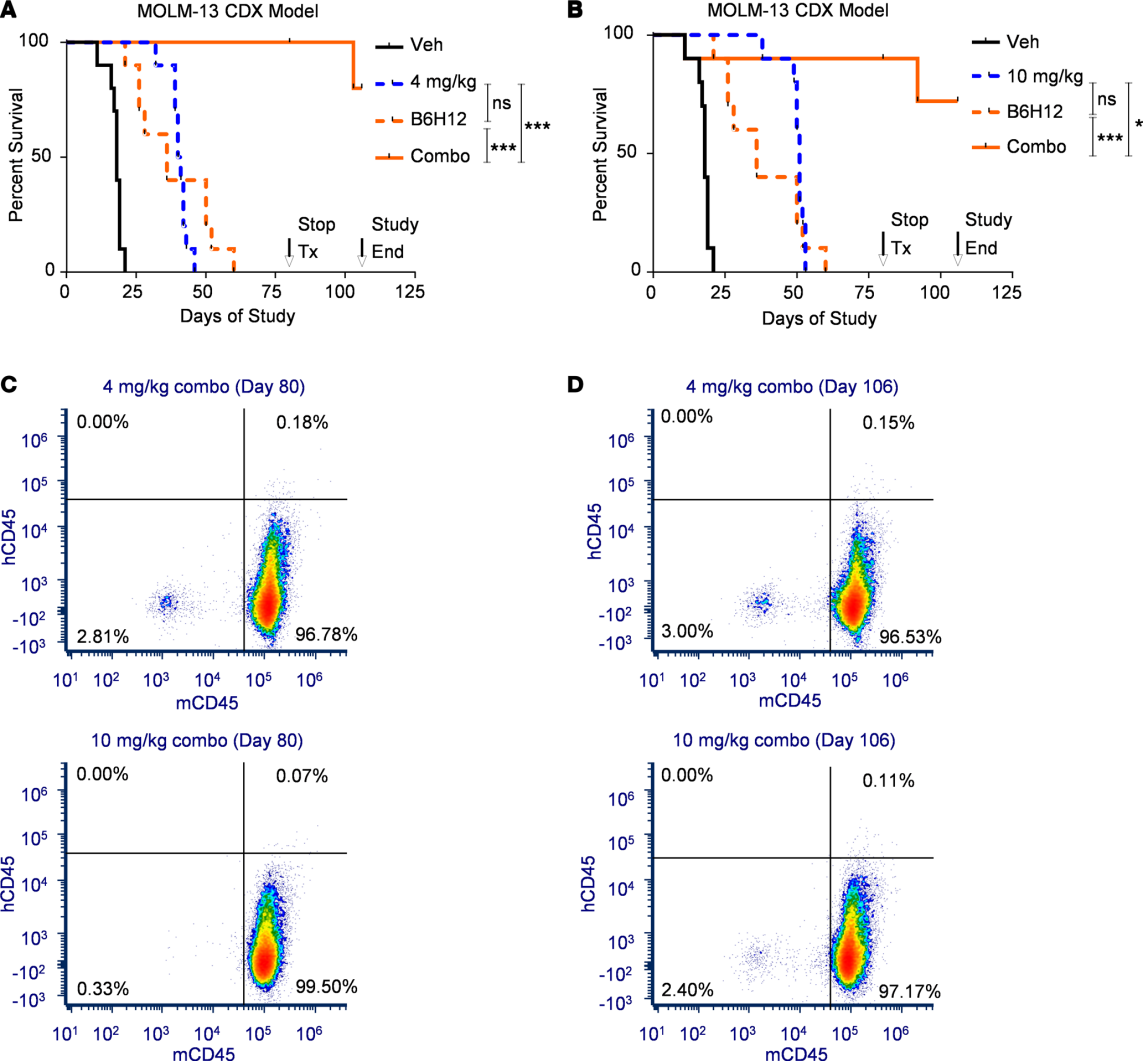
HOSU-53 in combination with anti-CD47 therapy results in long-term disease-free survival in the MOLM-13 CDX AML in vivo model. (**A**) Using the FLT3-mutant MOLM-13 CDX tumor–bearing model, 4 days after i.v. engraftment, NCG mice (*n* = 10/group) were enrolled to receive vehicle, daily p.o. 4 mg/kg of HOSU-53, i.p. 0.5 mg/animal daily for 21 days of CD47 antibody (clone B6H12, BioXCell), or combination of both. Arrows indicate stopping treatment on day 80 and end of study on day 106. In the combination arm, 5 of 10 mice were euthanized for bone marrow collection. On end of study, day 106, the surviving 4 mice were euthanized for bone marrow collection. Adjusted FDR *P* value ***≤0.0001. (**B**) Using the FLT3-mutant MOLM-13 CDX tumor–bearing model, 4 days after i.v. engraftment, NCG mice (*n* = 10/group) were enrolled to receive vehicle, daily p.o. 10 mg/kg of HOSU-53, i.p. 0.5 mg/animal daily for 21 days of CD47 antibody (clone B6H12, BioXCell), or combination of both. Arrows indicate stopping treatment on day 80 and end of study on day 106. In the combination arm, 4 of 10 mice were euthanized for bone marrow collection. On end of study, day 106, the surviving 4 mice were euthanized for bone marrow collection. Adjusted FDR *P* value *<0.05, ***≤0.0001. **A** and **B** share the vehicle and CD47 antibody arms. (**C**) Representative flow cytometry plot demonstrating the lack of residual human CD45 in bone marrow samples harvested at day 80, end of treatment. (**D**) Representative flow cytometry plot demonstrating the lack of residual human CD45 in bone marrow samples harvested at day 106, end of study.

We observed that CD47 expression was also increased with DHODHi treatment, using both the MV4-11 AML cell line and primary patient AML cells. As higher expression of CD47 on AML leukemia stem cells promotes AML immune evasion from phagocytes and correlates with worse overall survival (45), this increase in CD47 suggests an intrinsic protective response to DHODHi. This CD47 upregulation was DHODHi dependent, as suggested by reversal with uridine treatment (Supplemental Figure 8). Therefore, we determined if these DHODHi-treated cells were more prone to phagocytosis with blockade of the CD47/SIRP-α interaction. Generation of murine bone marrow–derived macrophages coincubated with DMSO-treated AML cells demonstrated minimal phagocytosis with isotype control (Supplemental Figure 9). There was an increase in phagocytosis with CD47 blockade alone using the CD47-blocking antibody B6H12. However, 72-hour HOSU-53 treatment of AML cells greatly enhanced phagocytosis in the presence of the B6H12 antibody, with evidence of multiple target cells being phagocytosed by a single macrophage (Supplemental Figure 9B). To determine if a similar observation occurred in vivo, we utilized the MOLM-13 CDX model to investigate the outcome of HOSU-53 combination with anti-CD47, B6H12. The 4 mg/kg HOSU-53 and B6H12 anti-CD47 monotherapy arms achieved modest but significant survival benefit while 10 mg/kg HOSU-53 performed well as in previous studies. However, the combination arms resulted in impressive, prolonged survival, which we have not observed with our prior combination regimens with this aggressive xenograft model (Figure 8, A and B). Whereas all mice in the vehicle and single agent groups had met ERC between 22 and 55 days, all animals in the combination groups remained on the study at day 80. Therefore, we stopped treatment and sacrificed 4–5 mice per combination arm for bone marrow isolation and kept the remaining mice for an additional ~25 days (without treatment) to determine if any residual disease would relapse. At day 80, hCD45 was not detected in the harvested bone marrow cells (Figure 8C). Similarly, at the end of the study (day 106), hCD45 was not detected in the bone marrow cells harvested from the surviving mice (Figure 8D), verifying lack of detectable disease when therapy was stopped till end of the study.

## Discussion

To date, outside of bone marrow transplant, curative strategies in AML are rare, with the exception of the transformative use of ATRA differentiation therapy–based regimens in APL, which cures a large majority of APL cases. The success of this approach indicates the impact differentiation therapies can have in AML treatment. The identification that pyrimidine depletion promotes differentiation independent of a certain AML mutation prompted us to focus on developing a clinically viable inhibitor of this pathway.

Herein, we describe HOSU-53, a potent DHODHi with in vivo efficacy superior to other DHODH inhibitors and exceptional pharmaceutical properties for clinical advancement. HOSU-53 was generated through detailed analysis of structure/activity relationship of more than 150 analogs. It demonstrates potent in vivo antileukemic activity in AML as monotherapy and in combination with other approved AML therapeutics, including the FLT3 inhibitor, gilt, and HMAs, decitabine and aza. Notably, HOSU-53 with decitabine has superior activity in a TP53-deficient AML model, suggesting activity in the most difficult-to-treat subtype, TP53-mutant AML.

We provide evidence for several translational opportunities to develop this class of therapeutics, including the identification of the biomarker (DHO) that distinguishes therapeutic efficacy from prohibitive toxicity. Unlike many other biomarkers used in drug development, plasma DHO is easy to measure and quantify and could be incorporated into early human trials exploring dose optimization. The development of potent DHODHi has been limited by expected on-target toxicities of hematopoietic cell suppression and gastrointestinal symptoms, such as diarrhea and stomatitis (25, 26). We have demonstrated how the accumulation of DHO correlates with the survival advantage in our in vivo work, and higher levels of DHO are predictive of toxicity when increased concentrations of HOSU-53 are given. We hypothesize that this directly measurable pharmacodynamic biomarker that correlates with both efficacy and toxicity will facilitate development of HOSU-53 by enabling us to select the optimal dose of this therapeutic and in real time predict individuals who may require a dose reduction. This assay can be performed in relatively real time, offering the opportunity to effectively navigate the therapeutic index of HOSU-53.

In our PDX study, in addition to corroborating enhanced benefit with aza combination, when we further assessed the morphology of cells in the HOSU-53 mice, we found an abundance in mature myeloid cells, suggesting the reprograming of AML cells toward functional cells. Antibody-mediated effector mechanisms offer a great avenue for applying immunotherapies in AML, particularly if combined with agents that could mediate immune function. While combining HOSU-53 with other traditional AML therapeutics is attractive, combination with therapeutic antibodies represents an unexplored path to avoid the potential overlap of toxicity. Treatment with HOSU-53 enhanced *E*. *coli* phagocytosis, corroborating an activation in innate immune function. We believe this to be, in part, a result of the differentiation properties of HOSU-53, prompting us to explore synergy with antibody therapies.

In both AML cell lines and primary AML cells, we demonstrate HOSU-53 increases CD38 expression on the cellular surface. To date, activity of CD38 antibodies as monotherapy or as adjuvant to donor lymphocyte infusion has been explored in AML (NCT03067571, NCT03537599) (46), though final results have not yet been published. Our in vivo data with anti-CD38 therapies would support the combination of HOSU-53 and CD38-directed immunotherapies, providing a superior survival advantage in vivo, verifying translational relevance for the combination of DHODHi with anti-CD38 therapies. Other groups have demonstrated that ATRA can promote synergy with CD38 antibodies by promoting AML blast fratricide (i.e., AML cells targeting one another). Given the diminished functionality of macrophages in the in vivo immunocompromised system examined, it is assumed this fratricide mechanism underlies the HOSU-53 and CD38-targeted antibody synergy.

In addition to modulating CD38 expression on AML cells, we demonstrated that HOSU-53 also promotes surface upregulation of CD47. CD47 is an antiphagocytosis immune checkpoint molecule, which mediates tumor evasion via the CD47/SIRP-α interaction, resulting in a “do not eat me” signal (45, 47). To determine if this represents an adaptive response suggesting vulnerability to phagocytosis, we examined untreated and HOSU-53–treated AML cells together with the B6H12 antibody that interferes with binding of SIRP-α on murine macrophages with tumor CD47. Notably, we observed increased murine macrophage phagocytosis with HOSU-53–treated cells. This prompted an in vivo study with HOSU-53 together with B6H12 where we saw compelling survival prolongation in an AML xenograft model with cure of a large subset of animals. Similar to that observed with CD38 therapeutic antibodies, but more potent with CD47 blockade, we hypothesize that the mechanism of clearance is fratricide. These results suggest considerable promise for combination of HOSU-53 or other DHODHi together with CD47 antibodies. In vivo studies using both alternative xenograft models and immunocompetent murine models are planned to further investigate the outcome of this combination in the presence of a functional immune system.

In summary, our preclinical assessment for HOSU-53 provides strong rationale for clinical development in AML both as monotherapy and in combination with other targeted small molecule therapy and immune-based therapies, particularly with CD47 blockade, resulting in compelling curative potential in the AML disease model. For the success of DHODHi in the clinic as a monotherapy and learning from past BRQ clinical trials, we believe that pharmacodynamic monitoring of plasma DHO levels can be used to predict tolerable versus toxic doses, which is an important factor as this class of drugs is developed in AML.

## Methods

### Sex as a biological variable

Male and female mice have been used, and each study description below states the sex of mice used.

### Enzyme inhibition of human DHODH

Cell-free hDHODH (amino acid residues 31–395) enzyme inhibition assays were performed at Reaction Biology. Details are described in the Supplemental Methods.

Single-dose HOSU-53 PK and PD modeling HOSU-53 was prepared in 40% (w/v) 2-hydroxypropyl β-cyclodextrin (HPβCD) in water and administered either as a single dose of 3 mg/kg (dose concentration = 0.6 mg/mL, dose volume = 5 mL/kg) via i.v. injection or as 10 mg/kg p.o. (dose concentration = 1 mg/mL, dose volume = 10 mL/kg) using male C57BL/6 mice (*n* = 3/arm) (Charles River Laboratories). In the i.v. cohort, serial blood samples were collected in tubes containing K2EDTA at 0.083, 0.25, 0.5, 1, 2, 6, 24, 48, and 72 hours postdose while in the oral gavage cohort, serial blood samples were collected at 0.25, 0.5, 1, 2, 4, 8, 24, 48, and 72 hours postdose. Blood samples were processed to plasma to analyze for HOSU-53 and l-dihydroorotic acid (DHO) concentrations using liquid chromatography-tandem mass spectrometry.

### Cell lines and culture media

MOLM-13, OCI-AML3, and MV4-11 cells were purchased from the Leibniz Institute DSMZ-German Collection of Microorganisms and Cell Cultures. THP-1, U937, and Kasumi-1 cell lines were purchased from the American Type Culture Collection. Cells were cultured in RPMI 1640 with 10% fetal bovine serum (FBS), 2 mM l-glutamine, 56 U/mL penicillin, and 56 μg/mL streptomycin (Thermo Fisher Scientific) and incubated at 37°C and 5% CO_2_. Cell lines were routinely tested for mycoplasma. Short tandem repeat testing was routinely performed for authentication.

### In vitro proliferation assay

A total of 10,000–20,000 cells from different AML lines or 50,000–75,000 cells of primary AML blasts were treated in a 96-well plate for 72 hours or 96 hours at 37°C, 5% CO_2_. CellTiter 96 Aqueous MTS reagent (Promega) was added to cells followed by a 3-hour incubation at 37°C, 5% CO_2_. The absorbance was measured at 490 nm using DTX 880 plate reader, and the IC_50_ was calculated using GraphPad Prism.

### In vitro uridine rescue assay

In vitro analysis of the antagonistic activity of uridine to HOSU-53 efficacy on MOLM-13 cells was done using MTS proliferation assay with increasing concentrations of uridine, HOSU-53, or the combination. Three biological replicates were analyzed using Combenefit software with mathematical models for highest single agent (48).

### Primary AML in vitro culture

Cells were thawed in 50% FBS RPMI and then cultured in StemSpan (StemCell Technologies) media supplemented with human FLT3L, SCF, GM-CSF, G-CSF, IL-3, IL-6, TPO, and EPO cytokines for 7 days (LTC).

### E. coli phagocytosis assay

The THP-1 AML cell line was treated for 6 days, then washed and cocultured with pHrodo Deep Red E. coli BioParticles (P35360, Invitrogen) per the manufacturer’s protocol. Ten-minute pretreatment with 10 μM of cytochalasin D (actin polymerization inhibitor) was used as a negative control for phagocytosis. After 90 minutes of incubation with *E*. *coli*, cells were washed and run on the Cytek Aurora spectral flow cytometer. This assay has been demonstrated to represent an exceptional pharmacodynamic model of differentiation in AML (30).

### Uridine plasma concentration quantification

Plasma from blood and bone marrow samples was obtained following appropriate dosing administration and saved in the –80°C freezer till the time of analysis. Details are described in the Supplemental Methods.

### Flow cytometry

After 72 hours or 7 days of treatment of DHODHi, cells were collected and washed with PBS, blocked with human Fc block (BioLegend), and stained with antibodies for 30 minutes on ice. Antibodies were washed off and cells resuspended in FACS buffer (2% FBS, 2 mM NaN_3_, Dulbecco’s PBS) for analysis using BD LSRFortessa or Cytek Aurora spectral flow cytometer. Live/dead exclusion was done using LIVE/DEAD Fixable Near-IR (Invitrogen). All antibodies were purchased from BioLegend except CD47, which was purchased from BD Biosciences: CD11b clone M1/70, CD38 clone HIT2, CD14 clone M5E2, CD47 clone B6H12, CD33 clone WM53, CD3 clone UCHT1, CD19 clone HIB19, and CD45 clone HI30.

### In vivo murine studies

Several disseminated AML CDX studies were performed with validated cell lines using IACUC-compliant methods for engraftment and overall survival. GraphPad Prism was used for graphical presentations, and statistical analyses are described in detail in Statistics.

### MOLM-13 disseminated CDX model

#### Multiple ascending dose study.

Male 8- to 12-week-old NCG mice (NOD-Prkdc^em26Cd52^Il2rg^em26Cd22^/NjuCrl, Charles River) were engrafted with 1 × 10^5^ cells via tail vein i.v. injection. Four days after engraftment (day 1 of treatment), mice (*n* = 10/arm) were randomized to receive p.o. qd vehicle (40% HPβCD) or ascending doses of oral HOSU-53. The 0.1, 1, and 10 mg/kg arms received p.o. qd HOSU-53 while the 30 mg/kg arm was given for 13 days where weight loss was observed and subsequently subjected to a dosing holiday till day 18, when treatment was resumed with daily 20 mg/kg till moribundity or end of study, day 74. The 100 mg/kg cohort received treatment for 6 days, then showed dose-limiting toxicities, and treatment was halted.

#### Efficacy and PK/PD correlative study in MOLM-13 tumor–bearing mice.

Male 8- to 12-week-old NCG mice were engrafted with 1 × 10^5^ cells via tail vein i.v. injection. Four days after engraftment (day 1 of treatment), mice (*n* = 9–10/arm) were randomized to receive p.o. qd vehicle (40% HPβCD) or p.o. qd HOSU-53 at 4, 10, and 20 mg/kg or 30 mg/kg biwk till moribundity or end of study, day 73. An additional study was done for PK/PD correlation with the survival study where 9 mice per group were randomized to receive p.o. qd vehicle (40% HPβCD) or p.o. qd HOSU-53 at 4, 10, and 20 mg/kg or 30 mg/kg biwk till day 14. Mice were bled for plasma collection to analyze HOSU-53 and DHO concentrations 1 day after the first dose and on day 14 for cumulative plasma concentration analysis. Figure 3B and Figure 4, A and B, share the same vehicle group.

#### HOSU-53 and BAY matched-dose comparison.

Male 8- to 12-week-old NCG mice were engrafted with 1 × 10^5^ cells via tail vein i.v. injection. Four days postengraftment (day 1 of treatment), mice (*n* = 9–10/arm) were randomized to receive p.o. qd vehicle (40% HPβCD), HOSU-53, or BAY at 4 and 10 mg/kg till moribundity or end of study, day 73.

#### HOSU-53 synergy with aza HMA in MOLM-13 tumor–bearing mice study.

Female 8- to 12-week-old NCG mice were engrafted with 1 × 10^5^ cells via tail vein i.v. injection. Four days postengraftment (day 1 of treatment), mice (*n* = 10/arm) were randomized to receive p.o. qd vehicle (40% HPβCD, *n* = 10; or 60% Phosal 50 PG, 30% PEG 400, and 10% ethanol, *n* = 10), 4 mg/kg HOSU-53, 25 mg/kg ven, or i.p. 1.5 mg/kg aza on a 5 days on/16 days off cycle, combinations of double regimens and triple regimens till moribundity or end of study, day 53. The 2 vehicle groups are shown combined (*n* = 20 total).

#### HOSU-53 synergy with FLT3 inhibitor, gilt.

Male 8- to 12-week-old NCG mice were engrafted with 1 × 10^5^ cells via tail vein i.v. injection. At 10 days after engraftment (day 1 of treatment), mice (*n* = 10/arm) were randomized to receive p.o. qd vehicle (50/50 mix of 20% HPβCD and 0.5% methylcellulose + 0.2% Tween 80), 4 mg/kg HOSU-53, 30 mg/kg gilt, or their combination till moribundity or end of treatment, day 79. Treatment was predetermined to stop on day 79, after which any remaining mice would be monitored until ERC.

#### HOSU-53 synergy with anti-CD38 immunotherapy.

Male 8- to 12-week-old male NCG mice were engrafted with 1 × 10^5^ cells via tail vein i.v. injection. Four days after engraftment (day 1 of treatment), mice (*n* = 8/arm) were randomized to receive p.o. qd and i.p. vehicle (p.o. qd 40% HPβCD and biwk i.p. PBS), daily oral 4 mg/kg BAY or 10 mg/kg HOSU-53, 1 mg/kg i.p. dara biwk, or DHODHi combination with dara, 2.5 mg/kg i.p. isa biwk, or DHODHi combination with isa till moribundity or end of study, day 79. Figure 7, B and C, and Supplemental Figure 7, A and B, share 2 combined vehicle groups (*n* = 16 total).

#### HOSU-53 synergy with anti-CD47 immunotherapy.

Male 8- to 12-week-old NCG mice were engrafted with 1 5 10^5^ cells via tail vein i.v. injection. Four days after engraftment (day 1 of treatment), mice (*n* = 10/arm) were randomized to receive p.o. qd and i.p. vehicle, daily oral 4 mg/kg or 10 mg/kg HOSU-53, 0.5 mg/animal i.p. anti-CD47 B6H12 (BioXCell) daily for 21 days, or HOSU-53 combination with anti-CD47. At day 80, half of the surviving mice in the combination cohorts were euthanized for bone marrow collection, leaving the remaining mice in the group on study without receiving any treatment. At end of study at day 106, surviving mice were euthanized for bone marrow collection to confirm the presence or lack of hCD45 levels. Figure 8, A and B, share the same vehicle and anti-CD47 cohorts.

### HL-60 disseminated CDX model: HOSU-53 synergy with decitabine HMA

Female 8- to 12-week-old female NCG mice were engrafted with 1 × 10^7^ cells via tail vein i.v. injection. At 14 days after engraftment (day 1 of treatment), mice (*n* = 10/arm) were randomized to receive p.o. qd and i.p. vehicle (p.o. qd 40% HPβCD and 4 on/10 off i.p. PBS), 10 mg/kg HOSU-53, i.p. 0.4 mg/kg decitabine on a 4-day on/10-day off cycle, or their combination till moribundity or end of study day 82.

### PDX model: HOSU-53 synergy with aza HMA

An r/r MLL/AF10 rearranged, therapy-related AML passaged PDX sample (CCHMC-2017-14) was used. NOD.Cg-*Rag1^tm1Mom^ Il2rg^tm1Wjl^* Tg(CMV-IL3,CSF2,KITLG)1Eav/J (NRGS) mice were obtained from The Jackson Laboratory and bred at the Cincinnati Children’s Hospital Medical Center Humanized Mouse Resource. Male 7- to 14-week-old male NRGS mice were engrafted with 5 × 10^5^ cells via tail vein i.v. injection. NRGS mice (*n* = 9–10/arm) were randomized on day 13 after engraftment to receive vehicle, 10 mg/kg HOSU-53 p.o. 5 days/week, 0.5 mg/kg aza i.p. 4 days/week for 4 weeks, or combination of aza and HOSU-53. On day 27 after engraftment, BMA was performed to assess disease burden by measuring the surface expression of hCD45 on live cells (7-AAD–negative cells) using flow cytometry.

### Statistics

The preclinical studies are designed using a balanced ANOVA model with 10 replicates in each combination. The normally distributed outcomes are compared using ANOVA (49) and are compared in any 2 groups using a statistical contrast. For survival data, we fit the Cox proportional hazards model and compare any 2 groups using a contrast. The survival differences are displayed using Kaplan-Meier curves. Because almost all individuals died in some preclinical studies, we also fit ANOVA model on the logarithm of the survival time and compare groups using a contrast, which is efficient. The parameter estimates and unadjusted and adjusted (for multiple comparisons) *P* values are provided. We report results as significant (symbolized by “*”) at *P* < 0.05, very significant (“**”) at *P* ≤ 0.001, and extremely significant (“***”) at *P* ≤ 0.0001. All calculations are performed with SAS statistical software (V9).

### Study approval

The approval for use of deidentified human samples was obtained from The Ohio State University Leukemia Tissue Bank, which holds an Honest Broker status. Animal studies performed at Cincinnati Children’s Hospital Medical Center (CCHMC) were performed according to CCHMC IACUC–approved protocol. Studies performed at Charles Rivers Discovery Services are accredited by the Association for Assessment and Accreditation of Laboratory Animal Care International, which assures compliance with accepted standards for the care and use of laboratory animals.

### Data availability

Values for the data shown are available in the Supporting Data Values Excel file.

## Author contributions

OAE contributed to designing research studies, methodology, conducting experiments, acquiring data, analyzing data, supervision, writing the original draft, and reviewing and editing subsequent drafts. SF, AM, DCV, MLJ, BS, JRL, ES, BC, SJO, and RS contributed to the methodology, investigation, and data analysis of select experiments and to reviewing and editing of the manuscript. SV, MA, and TEG contributed to the investigation and methodology of the chemistry and synthesis of HOSU DHODHi analogs and to reviewing and editing of the manuscript. JT contributed to the investigation of select in vivo experiments and to reviewing and editing of the manuscript. ASM, EC, ST, and SDB contributed to the methodology of select experiments and to reviewing and editing of the manuscript. ZX contributed to the investigation, methodology, and data analysis of the uridine level analysis and to reviewing and editing of the manuscript. MAP and CCC contributed to the methodology, data analysis, and visualization of PK and PD experiments and to reviewing and editing of the manuscript. CS and MW performed the PDX study. JE and J Patrick contributed to resource acquisition, funding acquisition, project administration, and reviewing and editing of the manuscript. JR, J Pan, and SNR contributed to the formal statistical analysis and to reviewing and editing of the manuscript. CEB, GH, EH, and JCB contributed to designing research studies, supervision, resources, data curation, formal analysis, funding acquisition, methodology, project administration, and reviewing and editing of the manuscript.

## Supplementary Material

Supplemental data

Supplemental table 1

Supplemental table 2

Supplemental table 3

Supplemental table 4

Supporting data values

## Figures and Tables

**Table 1:**
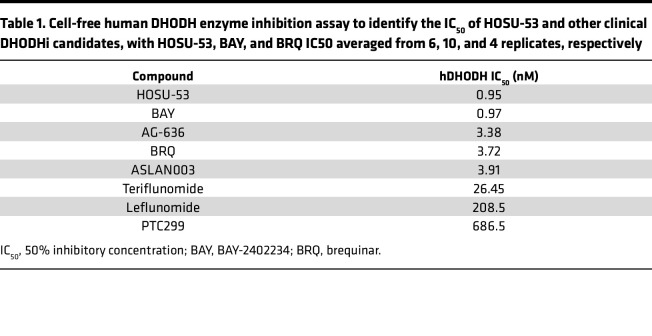
Cell-free human DHODH enzyme inhibition assay to identify the IC_50_ of HOSU-53 and other clinical DHODHi candidates, with HOSU-53, BAY, and BRQ IC50 averaged from 6, 10, and 4 replicates, respectively

**Table 2:**
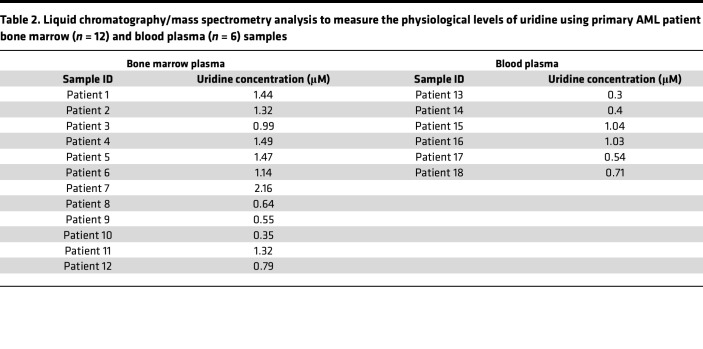
Liquid chromatography/mass spectrometry analysis to measure the physiological levels of uridine using primary AML patient bone marrow (*n* = 12) and blood plasma (*n* = 6) samples

**Table 3:**
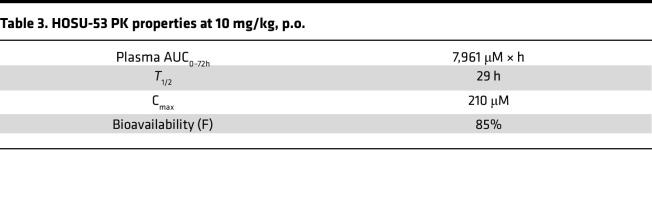
HOSU-53 PK properties at 10 mg/kg, p.o.

## Version 1 04/22/2024

Electronic publication
