# Supplementary material for: Pyrimidine depletion enhances targeted and immune therapy combinations in acute myeloid leukemia
Source: JCI Insight. 2024 Apr 22;9(8):e173646. doi: 10.1172/jci.insight.173646 (PMC11141866; doi:10.1172/jci.insight.173646)
Supplement: Supplemental data [file jciinsight-9-173646-s081.pdf]

Supplement Material

General procedures for preparation of HOSU-53

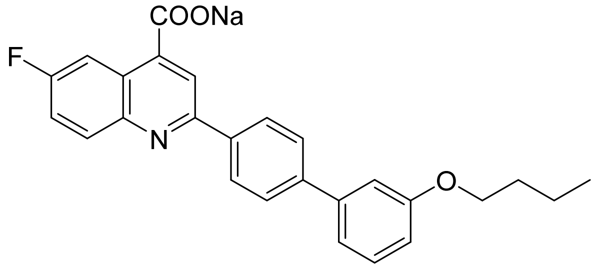

HOSU-53 Sodium salt

sodium 2-(3'-butoxy-[1,1'-biphenyl]-4-yl)-6-fluoroquinoline-4-carboxylate
Chemical Formula: $C_{26}H_{21}FNNaO_3$
Exact Mass: 437.1403
Molecular Weight: 437.4462

OR

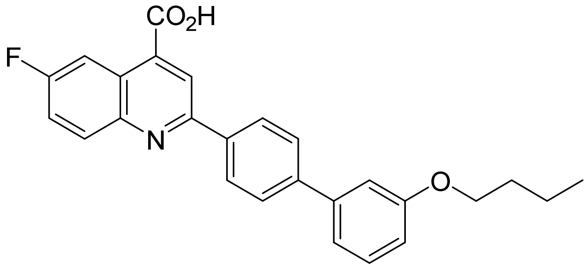

HOSU-53 free acid

2-(3'-butoxy-[1,1'-biphenyl]-4-yl)-6-fluoroquinoline-4-carboxylic acid
Chemical Formula: $C_{26}H_{22}FNO_3$
Exact Mass: 415.1584
Molecular Weight: 415.4644

General procedure for preparation of compound C1

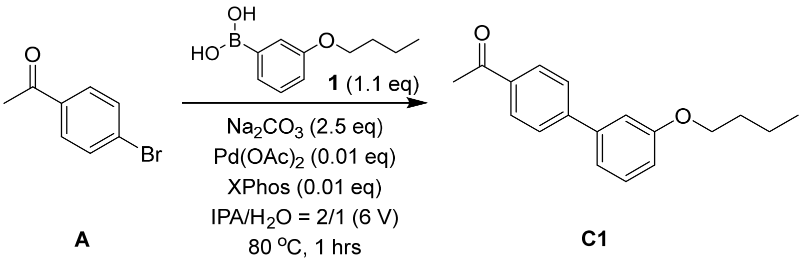

The mixture of compound **A** (40.0 g, 201 mmol, 1.00 eq) in IPA (200 mL) and H_2O (100 mL), compound **1** (42.9

g, 221 mmol, 1.10 eq), Na_2CO_3 (53.3 g, 502 mmol, 2.50 eq), $Pd(OAc)_2$ (451 mg, 2.01 mmol, 0.01 eq) and XPhos (958 mg, 2.01 mmol, 0.01 eq) was added to the mixture. The mixture was degassed and purged with N_2 for 3 times, and then the mixture was stirred at 80 °C for 1 hr under N_2 atmosphere. TLC (petroleum ether / ethyl acetate = 20 / 1, compound **A**: R_f = 0.61, compound **C1**: R_f = 0.43) indicated the compound **A** was consumed completely, and one major new spot with larger polarity was detected. The reaction mixture was diluted with H_2O (800 mL) and extracted with ethyl acetate (400 mL, 300 mL, 200 mL). The combined organic layers were washed with brine (600 mL), dried over Na_2SO_4, filtered and concentrated. The crude product was triturated with petroleum ether at 25 ºC for 4 hrs. The mixture was filtered and the filter cake was wash with petroleum ether (30.0 mL) and dry under reduced pressure. Compound **C1** (30.0 g, 109 mmol, 54.5% yield, 98% purity) was obtained as a off-white solid.

1H NMR of C1:

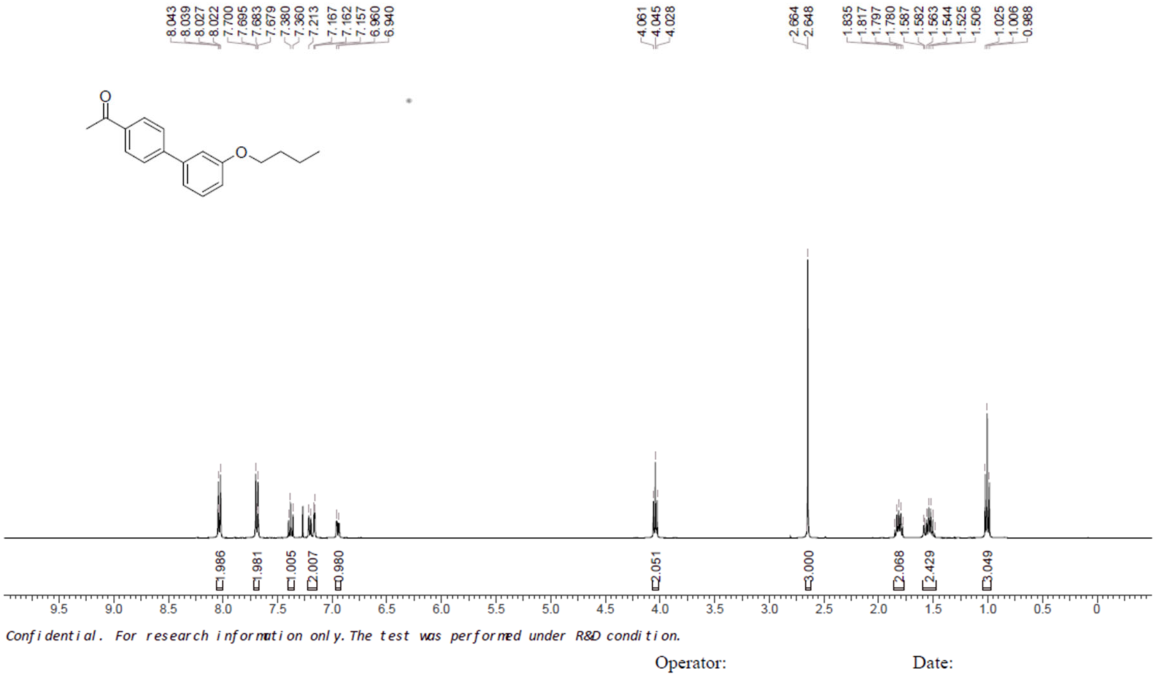

General procedure for preparation of compound HOSU-53 free acid.

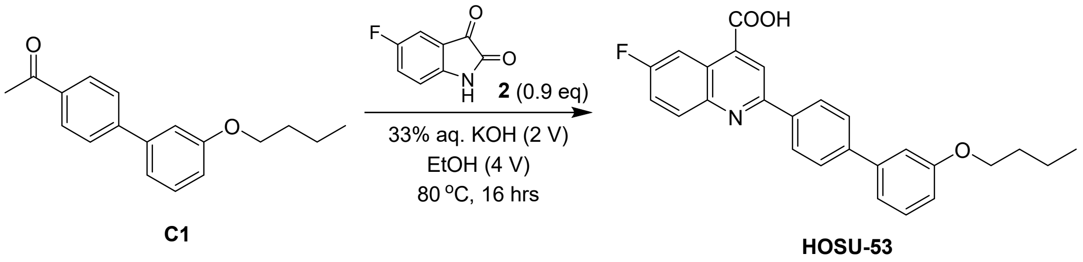

The mixture of compound **C1** (30.0 g, 112 mmol, 1.00 eq) in KOH (60.0 mL, 33% purity) was stirred and heated to 35 °C until clear yellow solution formed. Compound **2** (16.6 g, 101 mmol, 0.90 eq) and EtOH (120 mL) was added to this solution. The reaction mixture was stirring at 80 °C for 12 hrs. TLC (petroleum ether / ethyl acetate = 1 / 1, compound **C1**: $R_f = 0.57$, compound **HOSU-53**: $R_f = 0.35$) indicated compound **C1** was consumed completely, and one major new spot with larger polarity was detected. The reaction mixture was cooled to 25 °C. The pH was adjusted to 4 with aq. HCl (6 M). The mixture filtered under reduced pressure to give a residue. The crude product was triturated with petroleum ether / ethyl acetate = 3 / 1 (150 mL) at 20 °C for 12 hrs. The mixture was filtered and the filter cake was wash with petroleum ether (30.0 mL) and dry under reduced pressure. Compound **HOSU-53** (20.0 g, 48.1 mmol, 43.1% yield, 97% purity) was obtained as a yellow solid.

1H NMR of HOSU-53:

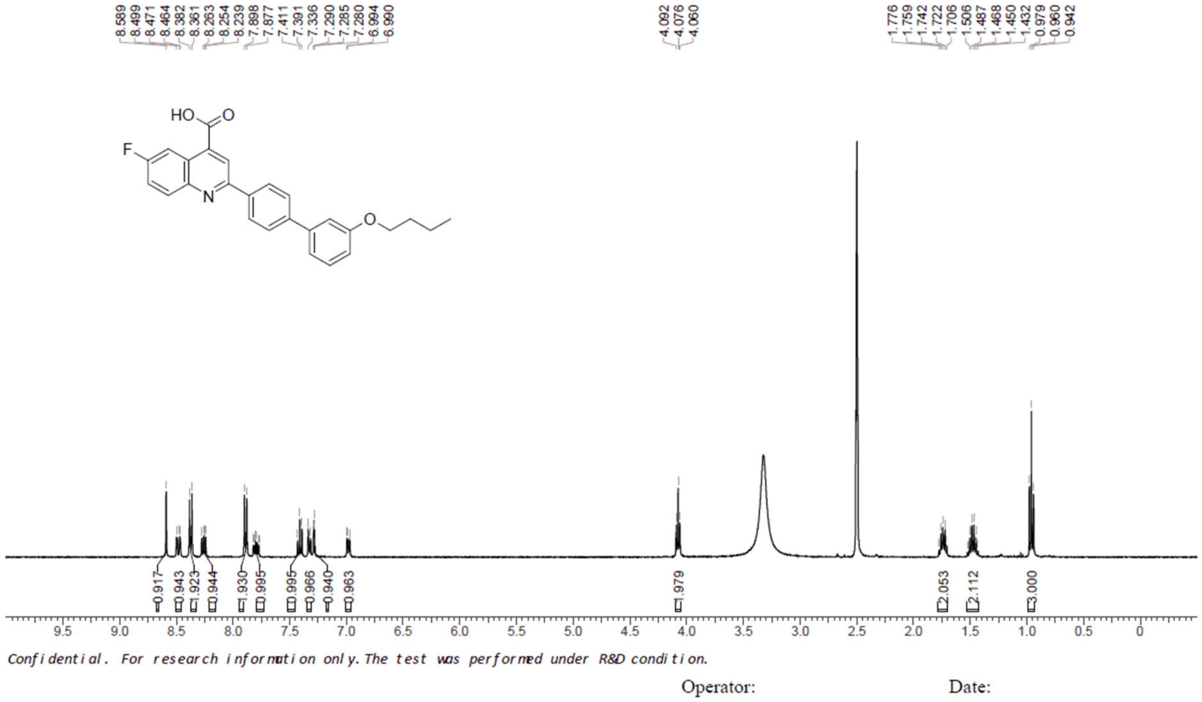

General procedure for preparation of compound HOSU-53 Sodium Salt

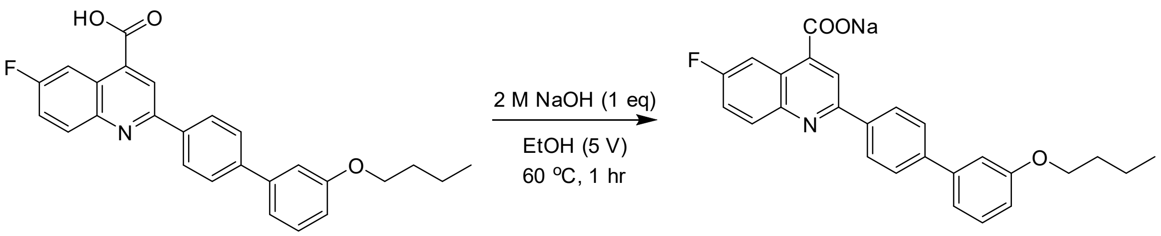

HOSU-53 **HOSU-53 Na salt**

To a solution of compound **HOSU-53 free acid** (18.0 g, 43.3 mmol, 1.00 eq) in EtOH (120 mL) was added aqueous NaOH (2 M, 21.7 mL, 1.00 eq). The mixture was degassed and purged with N_2 for 3 times, and then the mixture was stirred at 60 °C for 1 hr under N_2 atmosphere. The reaction mixture was concentrated under vacuum to removed EtOH. H_2O (250 mL) was added to the residue and the mixture was freeze-dried to give the product. ***HOSU-53 sodium salt*** (16.8 g, 38.4 mmol, 88.6% yield) was obtained as a yellow solid. MS $(M+1)^+$: calcd. m/z = 416.17, found m/z = 416.1.

1HNMR of HOSU-53 sodium salt:

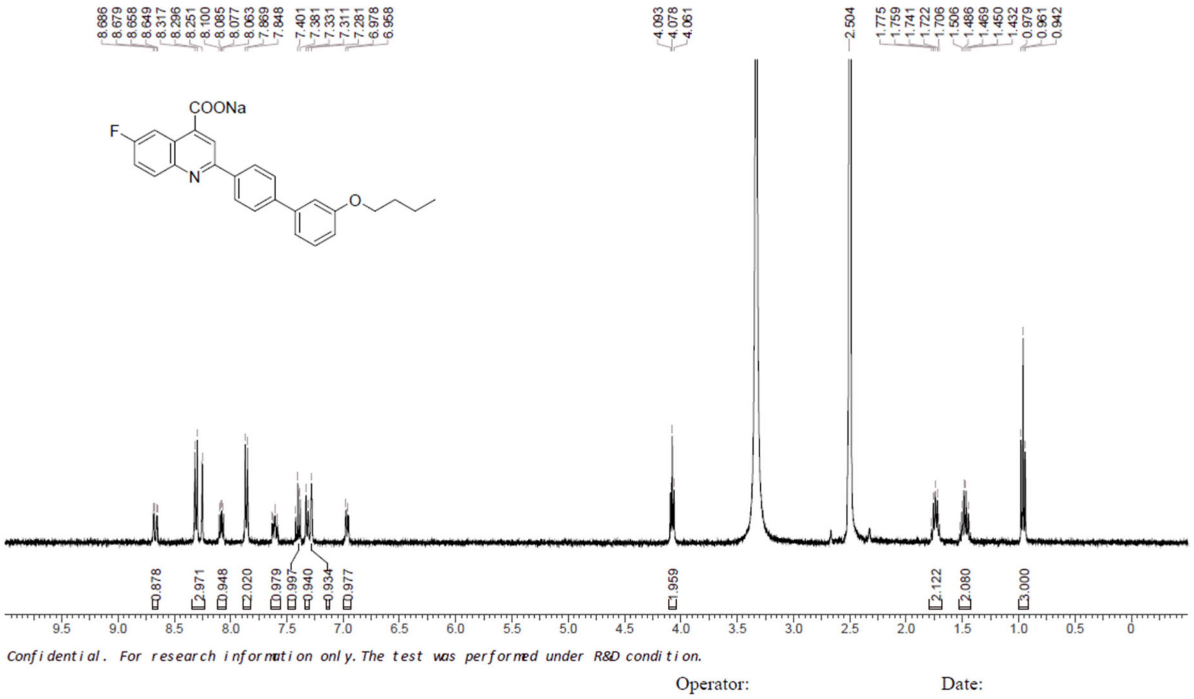

Enzyme inhibition of human DHODH

Cell-free *hDHODH* (amino acid residues 31-395) enzyme inhibition assays were performed at Reaction Biology (Malvern, PA, USA). Briefly, a two-step fluorescence-based assay was used to test compounds in duplicates using a 10-dose IC_{50} mode with 3-fold serial dilution starting at 1 μM or 10 μM. The enzymatic step for *hDHODH* (used at 5 nM per reaction) catalyzes the oxidation of L-DHO (used at 15 or 25 μM per reaction) to orotate and followed by resazurin (used at 60 or 80 μM per reaction) catalyzed to resorufin. Mixture is incubated for 45 minutes or one hour followed by the stop and detection step via orotate addition (used at 5 mM final concentration per reaction) to stop the reaction and detect the fluorescent signal from resorufin using Ex/Em=535/590 nm.

HOSU-53 concentration quantification in mouse plasma samples.

Plasma samples were obtained following appropriate dosing administration and saved in the -80℃ freezer till the time of analysis. Analyses by LC-MS/MS followed sample extraction by protein precipitation and separation by Ultra Performance Liquid Chromatography (UPLC, Waters Corporation, MA, USA) using a C18 column, 1.7um, 2.1x50mm, Mobile Phase A: 5:95:0.1 (v:v:v) Acetonitrile:Water:Formic Acid, Mobile Phase B: 50:50:0.1 (v:v:v) Methanol:Acetonitrile:Formic Acid. MS/MS analyses were performed on an API-6500 (Absciex MA, USA) in positive mode using an electrospray source at 550°C. The internal standard was d4-AEA monitored at 352.2 and 66.0 m/z transitions and the target analyte, HOSU-53, was monitored at 416.5 and 331.1 m/z transitions. The assay was qualified between 10-5,000 ng/ml HOSU53 to be within ±20% for QCs above the LLOQ in this range and ±25% at the LLOQ.

DHO concentration quantification in mouse plasma samples.

Plasma samples were obtained following appropriate dosing administration and saved in the -80℃ freezer till the time of analysis. Analyses by LC-MS/MS followed sample extraction by protein precipitation and separation by Ultra Performance Liquid Chromatography (UPLC, Waters Corporation, MA, USA) using a Phenomenex Aqua C18 100x4.6mm, 3uM, 125A, Mobile Phase A: 100:0.1 (v:v) Water:Formic Acid, Mobile Phase B: 100:0.1 (v:v) Methanol:Formic Acid. MS/MS analyses were performed on an API-4000 (Absciex MA, USA) in negative mode using an electrospray source at 550°C. The internal standard was labeled 13C14_L-Dihydroorotic Acid monitored at 161.3 and 117.1 m/z transitions and the target analyte, L-Dihydroorotic Acid (DHO), was monitored at 157.0 and 70.0 m/z transitions. The assay was qualified between 10.0-30,000 ng/ml DHO to be within ±20% for QCs above the LLOQ in this range and ±25% at the LLOQ.

Uridine concentration quantification in primary AML patient samples.

Plasma bone marrow samples were obtained following appropriate dosing administration and saved in the -80℃ freezer till the time of analysis. The sample preparation was conducted on ice, briefly, an aliquot of 10 μL plasma samples were spiked into 100 μL mobile phase solution containing 1000 ng/mL of internal standard (2-deoxyuridine, dU) followed by mixing with an equal volume of cooled (4℃) methanol, the mixture was vortexed for 30 sec and stored in -20℃ for 20 min, then centrifuged at 14,000 *g* at 4℃, followed by supernatant evaporation to dryness under nitrogen. The uridine calibration curve samples were prepared from intermediate stock solutions in methanol and in parallel with the patient plasma samples. The dried residues of both calibration curve sample and patient plasma samples were reconstituted in mobile phase for UPLC-MS/MS analysis.

Uridine and dU were separated on a 250 × 2.1 mm Hypersil Aquasil C18 column, which was coupled to an Aquasil pre-column, using the mobile phase consisting of 0.2 % formic acid in water at a flow rate of 0.2 ml/min with a Waters Acquity UPLC system. Pure methanol was then added to the flow at 0.2 ml/min and mixed post-column prior to the entrance to the ion source. The sample was introduced into the ion source at 20 μl/min after a 95:5 split. The mass spectrometer (Waters Quattro Premier) was operated under positive ion mode electrospray ionization and monitoring precursor/product ion pairs of m/z 229.1/112.9 for dU and m/z 245.2/113.0 for uridine. Data acquisition was performed using the MassLynx software. The assay was qualified with LLOQ of 5 ng/ml with the linear range from 5-1000 ng/ml.

Supplemental Figures

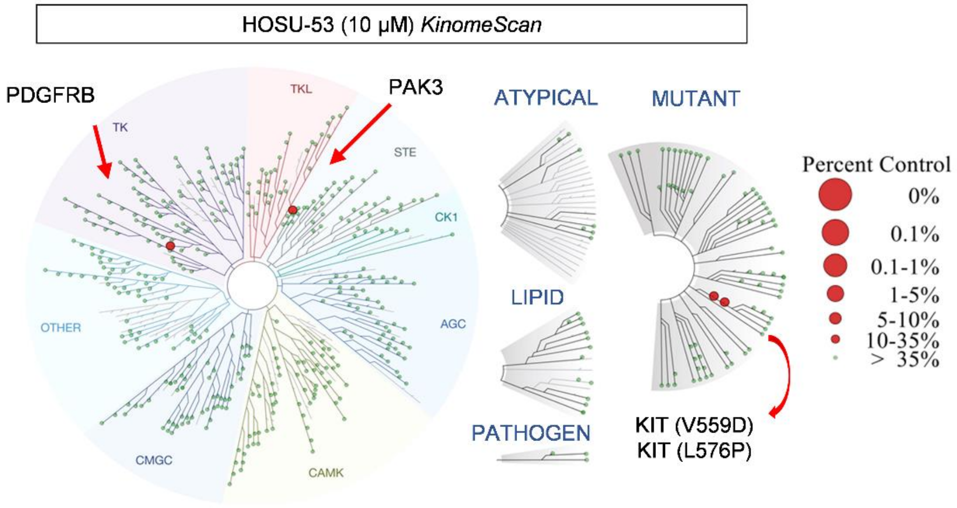

Supplemental Figure S.1. HOSU-53 KINOMEscan™ Profiling. Eurofins DiscoverX KINOMEscan® Profiling was conducted using 10 µM HOSU-53 to investigate the presence of any off-target kinase inhibition activity.

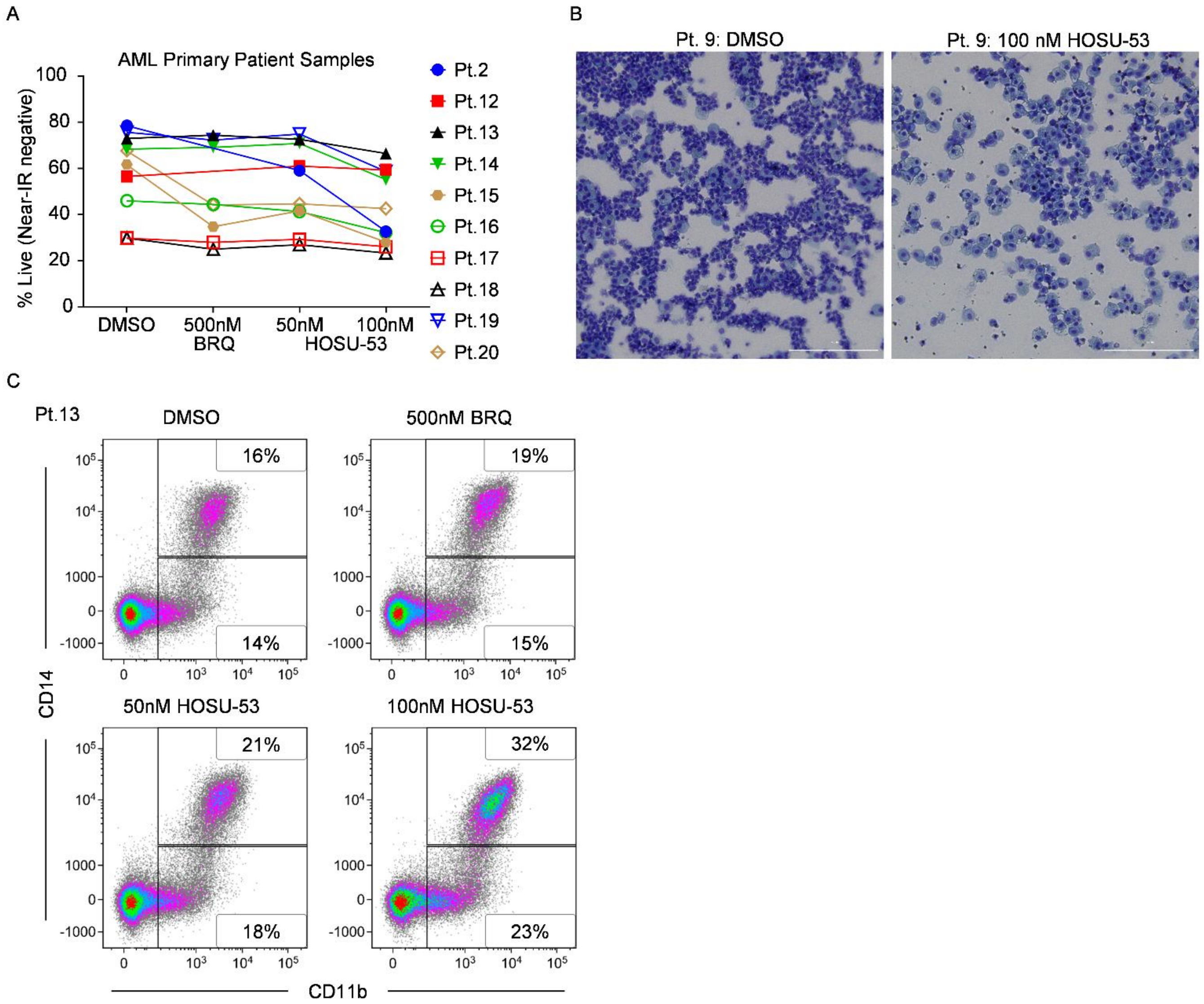

Supplemental Figure S.2. HOSU-53 shows in vitro potency and differentiation properties in AML. **A**). Flow cytometry analysis of viable primary AML patient samples (n=10) treated in vitro for seven days (long term culture, LTC). Live cells were measured using LIVE/DEAD™ Fixable Near-IR (NIR) gated on singlet cells. **B**) Representative hema 3TM differential staining (Fisher Scientific) of cytospin preparation slides for a primary AML sample treated with HOSU-53 in vitro for seven days (long term culture, LTC) to determine morphology changes. Images were taken using BioTek Cytation 5 Cell Imaging Multimode Reader at 20x magnification. **C**) Flow cytometry analysis of surface CD11b and CD14 expression using a primary AML patient sample (n=1) treated in vitro for seven days (long term culture, LTC). Cells were gated on live CD3-CD19- cells.

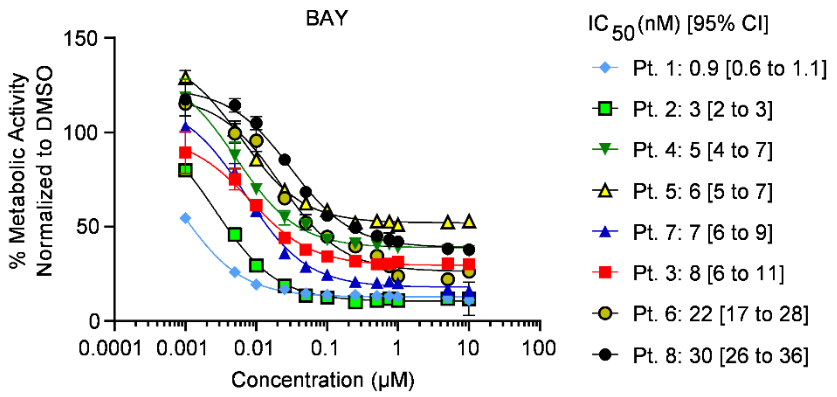

Supplemental Figure S.3. Bayer DHODHi, BAY 2402234 demonstrates in vitro potency in AML. MTS proliferation assay using primary AML samples (n=8) to determine the 50% inhibitory concentration (IC_{50}) of BAY after 96-hour treatment. GraphPad Prism was used to analyze, visualize, and calculate IC_{50} values.

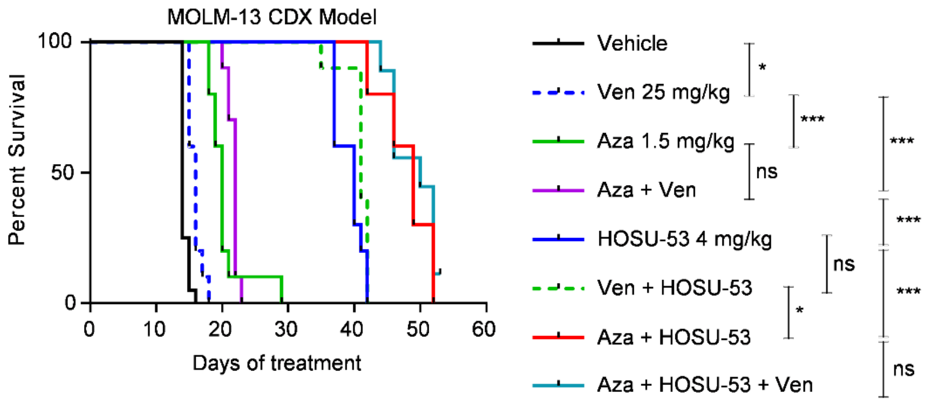

Supplemental Figure S.4. HOSU-53 significantly enhances azacitidine outcome. Using the FLT3 mutant MOLM-13 CDX tumor bearing model, four days post i.v engraftment, NCG mice (n=10/group) were enrolled to receive daily oral 4 mg/kg HOSU-53 to compare its monotherapy efficacy with daily oral 25 mg/kg venetoclax (ven) monotherapy or 1.5 mg/kg azacitidine (aza) HMA i.p. for five days every 16 days cycle monotherapy or the combination of aza/ven versus ven/ HOSU-53 versus aza/HOSU-53 versus the triple aza/ven/HOSU-53 regimen. One mouse in the Aza + HOSU-53 + Ven arm was excluded due to esophagus perforation non treatment related accident. Adjusted FDR p-value *<0.05, ***≤0.0001, nonsignificant (ns) ≥0.05.

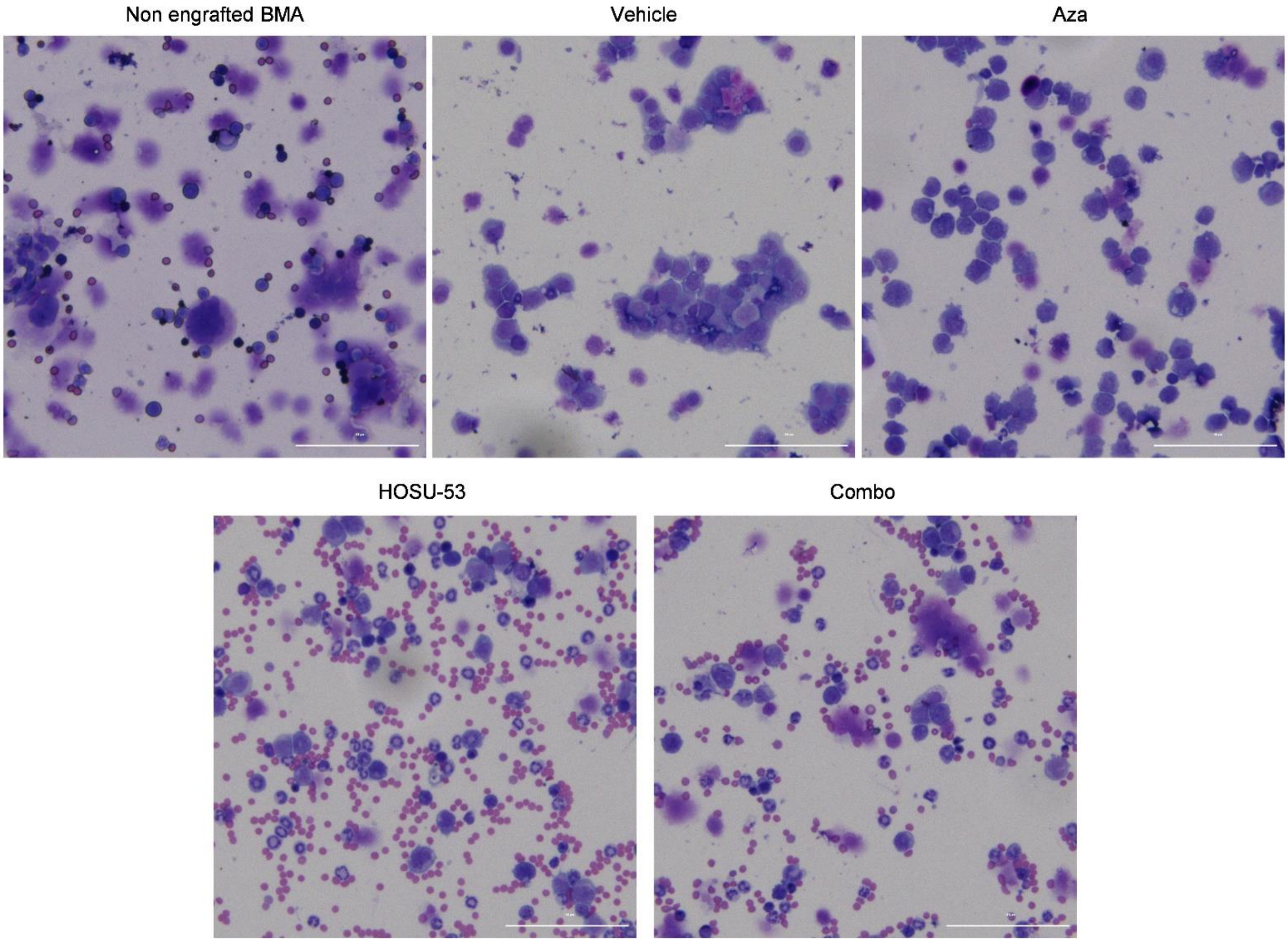

Supplemental Figure S.5. HOSU-53 treatment exhibits a differentiated cell morphology in a patient derived xenograft (PDX) in vivo tumor model. NRGS mice were treated with vehicle, 10 mg/kg HOSU-53 orally five days/week, 0.5mg/kg azacitidine (aza) i.p four days/week for four weeks, or combination of aza and HOSU-53. On day 27 post engraftment (D27), a bone marrow aspirate (BMA) was performed and cytospin slides were prepared for differential staining. Shown are representative Wright-Giemsa differential staining (Fisher Scientific) for the D27 BMA slides. Images were taken using BioTek Cytation 5 Cell Imaging Multimode Reader at 40x magnification.

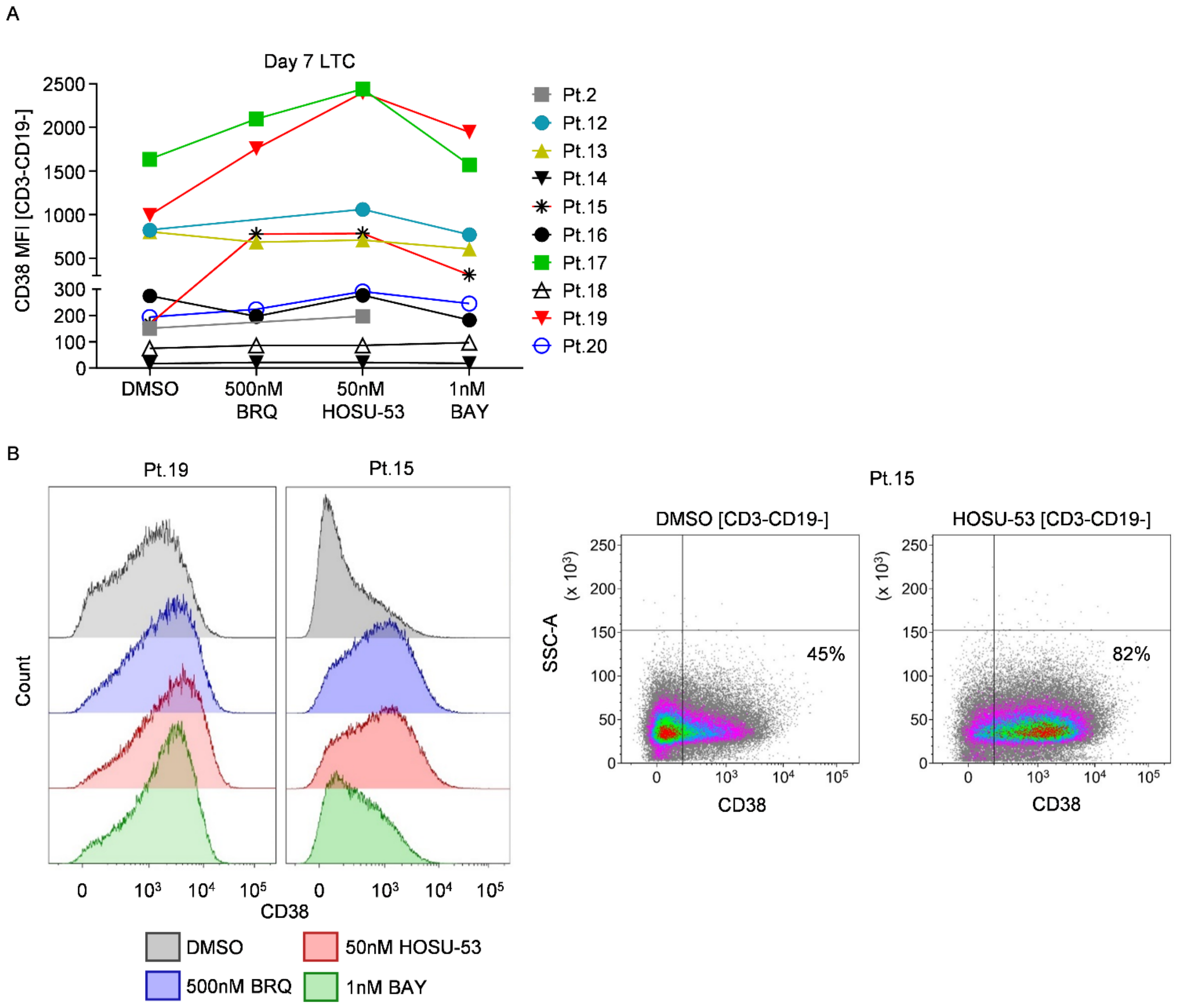

Supplemental Figure S.6. DHODHi modulates CD38 surface antigen expression. **A**) Flow cytometry analysis of CD38 surface expression in primary AML patient samples (n=8-10) treated *in vitro* for seven days (long term culture, LTC). Data is shown as mean florescent intensity (MFI) of CD38 gated on live CD45+CD3-CD19- cells, error bars indicate mean with 95% confidence interval. **B**) Representative flow cytometry overlay histogram plots for CD38 surface expression in primary AML patient samples demonstrating modulation of CD38 post DHODHi treatment.

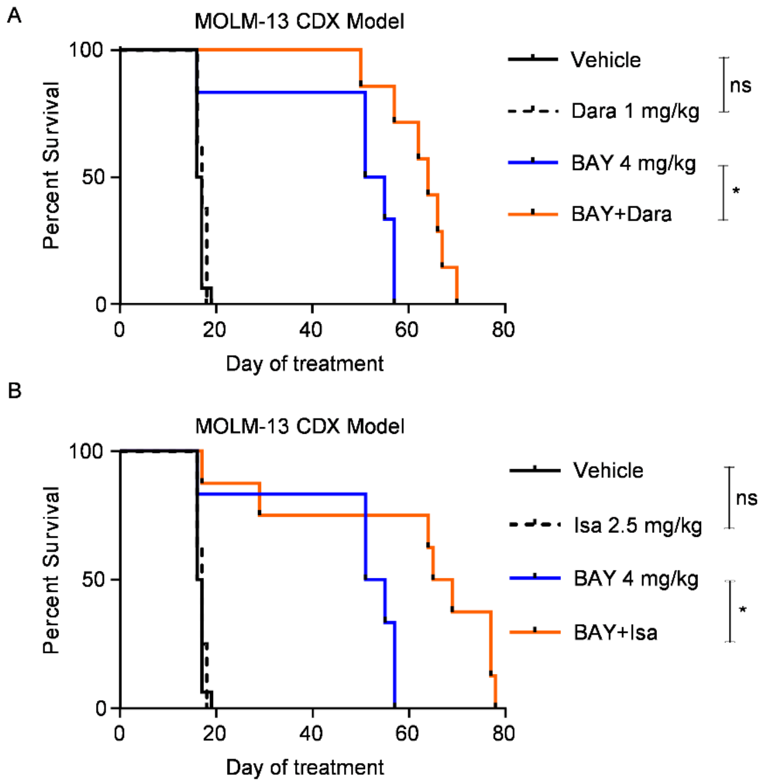

Supplemental Figure S.7. BAY DHODHi demonstrates synergy with anti-CD38 therapies. Using the FLT3 mutant MOLM-13 CDX tumor bearing model, four days post i.v engraftment, NCG mice (n=8/group) were enrolled to receive daily oral 4 mg/kg BAY or twice per week (biwk) 1 mg/kg i.p daratumumab (Dara) (**A**) or biwk 2.5 mg/kg i.p isatuximab, Isa (**B**) anti-CD38 antibodies or a combination of BAY and Dara (**A**) or combination of BAY and Isa (**B**). Treatment was stopped at day 80. Adjusted FDR p-value *<0.05, nonsignificant (ns) ≥0.05. In the 4 mg/kg BAY arm, two mice were excluded due to NTRu (non-treatment related unknown cause) and NTRa (non-treatment-related death due to accident, gavage error). In the 4 mg/kg BAY + Dara arm, one mouse was excluded due to esophageal perforation. Both Supplementary Figure S.7, A and B share the same vehicle and 4 mg/kg BAY cohorts and the vehicle, Dara, and Isa monotherapy arms in this figure are shared with Figure 7, B and C.

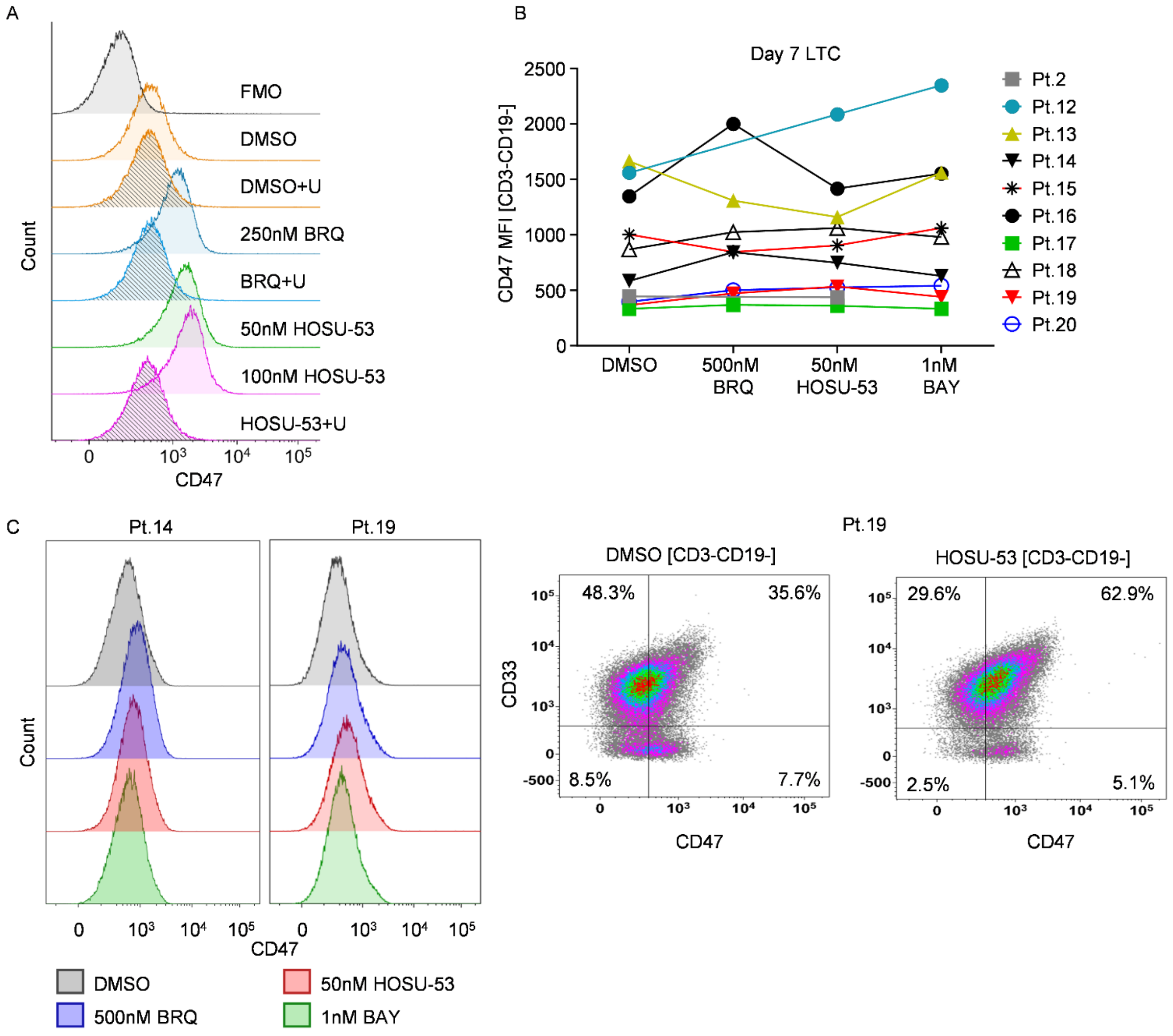

Supplemental Figure S.8. DHODHi modulates CD47 surface antigen expression. **A**) Representative flow cytometry overlay histogram plot for CD47 surface expression in MV4-11 cell line following a 72-hour treatment with BRQ or HOSU-53 in the presence or absence of 0.1mM uridine (U) supplementation (experiment was done five independent times). **B**) Flow cytometry analysis of CD47 surface expression in primary AML patient samples (n=8-10) treated in vitro for seven days (long term culture, LTC). Data is shown as mean florescent intensity (MFI) of CD47 gated on live CD45+CD3-CD19- cells, error bars indicate mean with 95% confidence interval. **C**) Representative flow cytometry overlay histogram plots for CD47 surface expression in primary AML patient samples demonstrating modulation of CD47 post DHODHi treatment.

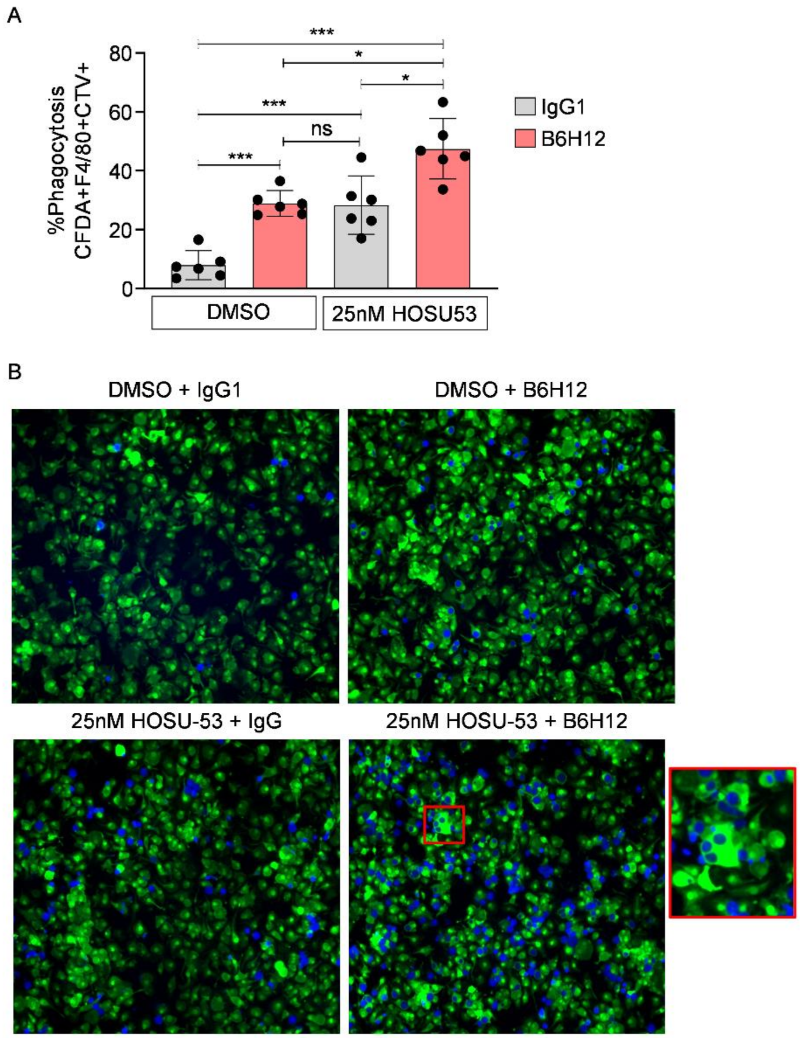

Supplemental Figure S.9. A) Murine bone marrow derived macrophages (BMDM, effector cells) were generated from harvested bone marrow cells grown in the presence of 20ng/ml murine M-CSF for 7-8 days where media was refreshed on day 3. After BMDM generation, they were collected and stained with CFDA-SE (green). MV4-11 AML target cells were pretreated for 72 h with DMSO or 10nM HOSU53 and stained with CTV (blue). Green BMDM cells were then co-cultured for 4 h with blue MV4-11 AML cells in the presence of 10 µg/ml isotype IgG1 or anti-CD47 antibody at 1:1 E:T ratio in an ultra-low binding tissue culture plate. Cells were then collected and analyzed using flow cytometry to quantify percentage of phagocytosed cells indicated by viable F4/80+CFDA+CTV+ cells (n=6). Adjusted FDR p-value *<0.05, ***≤0.0001, nonsignificant (ns) ≥0.05. **B**) Representative florescent microscopy images were taken using 20x objective to visualize antibody dependent cellular phagocytosis (ADCP). Prior to imaging, the wells were rinsed three time to remove MV4-11 target cells in suspension and allow the imaging of adherent effector cells (green BMDM) and residual blue MV4-11 target cells. The outlined area in the red box is manually enlarged.
